# Cheminformatics approach to identify andrographolide derivatives as dual inhibitors of methyltransferases (nsp14 and nsp16) of SARS-CoV-2

**DOI:** 10.1038/s41598-024-58532-7

**Published:** 2024-04-29

**Authors:** Jobin Thomas, Anupam Ghosh, Shivendu Ranjan, Jitendra Satija

**Affiliations:** 1 Centre for Nanobiotechnology (CNBT), Vellore Institute of Technology, Vellore, Tamil Nadu 632014 India; 2 NanoBio Research Lab, School of Nano Science and Technology, Indian Institute of Technology Kharagpur, Kharagpur, West Bengal 721301 India

**Keywords:** Covid-19, Andrographolide, Drug discovery, Natural compounds, nsp14, nsp16, Cheminformatics, Drug discovery and development, Screening

## Abstract

The Covid-19 pandemic outbreak has accelerated tremendous efforts to discover a therapeutic strategy that targets severe acute respiratory syndrome coronavirus 2 (SARS-CoV-2) to control viral infection. Various viral proteins have been identified as potential drug targets, however, to date, no specific therapeutic cure is available against the SARS-CoV-2. To address this issue, the present work reports a systematic cheminformatic approach to identify the potent andrographolide derivatives that can target methyltransferases of SARS-CoV-2, i.e. nsp14 and nsp16 which are crucial for the replication of the virus and host immune evasion. A consensus of cheminformatics methodologies including virtual screening, molecular docking, ADMET profiling, molecular dynamics simulations, free-energy landscape analysis, molecular mechanics generalized born surface area (MM-GBSA), and density functional theory (DFT) was utilized. Our study reveals two new andrographolide derivatives (PubChem CID: 2734589 and 138968421) as natural bioactive molecules that can form stable complexes with both proteins via hydrophobic interactions, hydrogen bonds and electrostatic interactions. The toxicity analysis predicts class four toxicity for both compounds with LD_50_ value in the range of 500–700 mg/kg. MD simulation reveals the stable formation of the complex for both the compounds and their average trajectory values were found to be lower than the control inhibitor and protein alone. MMGBSA analysis corroborates the MD simulation result and showed the lowest energy for the compounds 2734589 and 138968421. The DFT and MEP analysis also predicts the better reactivity and stability of both the hit compounds. Overall, both andrographolide derivatives exhibit good potential as potent inhibitors for both nsp14 and nsp16 proteins, however, in-vitro and in vivo assessment would be required to prove their efficacy and safety in clinical settings. Moreover, the drug discovery strategy aiming at the dual target approach might serve as a useful model for inventing novel drug molecules for various other diseases.

## Introduction

The outbreak of severe acute respiratory syndrome coronavirus-2 (SARS-CoV-2) in late 2019 led to a dramatic loss of human life at the global level and presented an unprecedented challenge to public health^1^. This highly contagious virus has undergone numerous mutations and to date, mankind is struggling to find a cure^2,3^. The Covid-19 vaccination helped in the management of the infection to some extent, but in the last two years, several reports have stated a significant increase in the cases of neurodegenerative and cardiovascular disorders associated with Covid-19 recovered patients^4^. The majority of these adverse effects are seen in the neurological system, with ischemic stroke, intracerebral hemorrhage, cerebral venous sinus thrombosis, transient ischemic attack and demyelinating diseases being the most frequent problems in cerebrovascular disorders^4^. Although these adverse effects are mild and temporary, in certain cases they can be serious and even fatal. Since the emergence of the pandemic, many researchers around the globe have been trying to find a cure, of which Paxlovid (main protease (M^pro^) inhibitor), Lagevrio (RNA polymerization disrupter) and Veklury (RNA-dependent-RNA-polymerase (RdRp) inhibitor) are approved by FDA (Food and Drug Administration) (fda.gov). These drugs are being administered to reduce the risk of hospitalization or death for patients (adults) with mild to moderate infection conditions. This indicates that the currently approved medications may only be beneficial for symptomatic relief and not very effective against the virus. Therefore, there is still a strong requirement for a specific therapeutic agent to combat SARS-CoV-2.

A multitude of SARS-CoV-2 proteins, such as the S protein^5^, main protease^6,7^, RNA-dependent-RNA-polymerase (RdRp), papain-like proteinase (PLpro), methyltransferases (MTs) and helicases^8^, have been discerned as prospective targets to discover a potent bioactive compound. Since structural proteins of SARS-CoV-2 are more prone to mutations, non-structural proteins (nsp) have gained more attention as protein targets for drug discovery^9–12^. Amongst these, MTs, specifically nsp 14 and nsp16, are the potential targets due to their vital role in viral RNA replication and evasion of host cell immune responses and negligible effect of mutations at conserved active site^9,12–14^. The MTs hold significant importance in the realm of anti-Covid-19 drug development, as these drugs serve as direct inhibitors of the nuclease proofreading activity and thus disrupt the viral replication and transcription. The MTs play a key role in the mRNA capping mechanism also. Particularly, the nsp14 is a bifunctional protein having exonuclease activity (proofreading 3′ to 5′) in the exon domain at the N-terminal region and a S-adenosyl methionine (SAM) dependent methyltransferase domain at the C-terminal. The N7-MTase enzyme specifically adds methyl group at the 7th nitrogen atom of the guanine moiety located at the 5ʹ end forming a cap-0 structure. In contrast, the nsp16 has only methyltransferase activity which specifically adds methyl group at the 2′-O position on the ribose sugar located at the 5′ terminus of the mRNA forming a cap-1 structure^10^. This mechanism stabilizes and protects the mRNA structure against degradation by host nucleases and facilitates the evasion from the host immune response. Therefore, it is a viable strategy to inhibit the viral MTs and thereby suppressing the viral replication and reducing the viral load in the infected host^15^. These unique functional characteristics along with biocatalytic action of methyltransferases make it a promising anti-viral drug target. To date, to the best of our knowledge, no specific drug is available against methyltransferases of SARS-CoV-2. Only a few compounds like amentoflavone, baicalin, daidzin, luteoloside, machaeriols and certain other synthesized compounds have been identified against this target protein which are also yet to be tested^16–18^. Further, no effort has been made to identify the potential drug candidate that can inhibit both nsp14 and nsp16 proteins which might offer synergistic therapeutic benefits to combat the SARS-CoV-2 disease.

Andrographolide (AGP), which is a diterpene lactone belonging to the isoprenoid family, is the most significant bioactive constituent of *Andrographis paniculate*^19^*.* This plant is routinely used in traditional medicinal practices for the treatment of various ailments including the common cold, diarrhoea, and fever resulting from various viral and non-viral origins, as well as for its potential as a health tonic^20^. The active constituents of this plant have been evidenced to have several drug-like properties against inflammation, cancer, obesity and diabetes^20^. Furthermore, AGP has been found to have better antiviral activities against various viral infections^21,22^. Recently, a few reports have demonstrated that andrographolide exhibits anti-SARS-CoV-2 activity by selectively targeting the host ACE2 receptor and other viral components, including spike protein, main protease, PL protease, 3-CL protease, and RdRp^15–18,23,24^. However, an in-depth investigation of the AGP and its derivatives against the SARS-CoV-2 is not explored yet. Further, to the best of our knowledge, no report is available on identification of AGP or its derivative towards the dual protein target to combat the viral diseases. Therefore, it is imperative to develop a highly effective natural medication candidate based on AGP derivatives that can specifically target the MTs protein with improved binding affinity and stability.

To date to the best of our knowledge, there is no established/approved potent drug molecule against the methyltransferases of SARS-CoV-2. Further, andrographolide derivatives have not been explored yet against the methyltransferases of SARS-CoV-2. For the first time, in the present study, we aim to target both nsp14 and nsp16 (dual target) with a common andrographolide derivative which is the first of its kind. To achieve the same, a comprehensive in silico investigation is conducted on 413 derivatives of andrographolide. These derivatives are subjected to virtual screening and molecular docking studies against both nsp14 and nsp16 target proteins to evaluate the binding affinity of drugs. The physicochemical properties, drug-likeness, and toxicity profiling are evaluated for the hit compounds which were further evaluated for their stability and reactivity through molecular dynamics (MD) simulations, Gibbs free energy calculations by employing MM-GBSA (molecular mechanics generalized born surface area) and DFT (density functional theory) calculations. These findings established two potent AGP derivatives having inhibition of both nsp14 and nsp16 proteins that can be used as potential natural anti-SARS-CoV-2 drugs.

## Materials and methods

### Retrieval and preparation of methyltransferase proteins

The crystal structures of the methyltransferase proteins, i.e. nsp14 and nsp16, were retrieved from the protein data bank (PDB) (rcsb.org) with PDB ID: 7R2V and 6WKQ respectively^25,26^. The 7R2V protein structure (1.98 Å resolution) contains the whole nsp14 protein sequence, i.e. C-terminal SAM-dependent methyltransferase domain and the N-terminal ExoN domain, with a total of 524 amino acid residues and a co-crystal SAH attached at the binding pocket of the protein. The protein structure was prepared by removing the ‘B’ chain (dimer subunit), SAH, TRS (2-amino-2-hydroxymethyl-propane-1,3-diol), PEG (di-hydroxyethyl)ether) and Zn^2+^ ions. Similarly, the 6WKQ protein structure (resolution of 1.98 Å) has sinefungin (SFG) as a co-crystal, which is a well-established inhibitor of MTs^11^. This protein structure was cleaned by removing the nsp10 domain in the structure along with SFG, formic acid, Zn^2+^ ion, and Na^+^ ion. Finally, both the protein structures were subjected to refinement and energy minimizations.

### Retrieval and preparation of andrographolide derivatives

A total of 413 AGP derivatives were retrieved from the PubChem database (using ‘similar structures search’ tool) which consisted of α, β-unsaturated γ-lactone as a common moiety. Before initiating virtual screening, all the compounds were energy minimized via Open Babel through Python programming^27^.

### Virtual screening

The virtual screening of the AGP derivatives against the MTs was accomplished using the Autodock Vina program via Python programming^28^. The grid box occupying the binding pocket residues of the methyltransferase proteins was created with an exhaustiveness value of 8. In the case of nsp14, the residues Asp352, Ala353, Tyr368, Trp385, and Cys387(SAH binding site)^29^ were selected for the grid box generation (15.178 × -8.265 × -19.955 Å as xyz coordinates) whereas in the case of nsp16, residues Asn6842, Asp6871, Asp6912, Asp6928, Gly6869, Gly6879, Gly7871, Tyr6930 and Cys6913 (SFG binding site)^30^ were selected (81.790 × 14.047 × 2.616 Å as xyz coordinates). The top ten AGP derivatives qualifying the cut-off binding energy value were selected for each protein.

### Molecular docking and ADMET profiling

To further refine and validate the virtual screening results, rigid molecular docking was carried out using AutoDock4 software^31^. Prior to molecular docking, the docking protocol was validated by superimposing the nsp14-SAH and nsp16-SFG complex obtained from PDB (PDB ID: 7R2V and 6WKQ respectively), onto the re-docked complex of co-crystal component (SAH/SFG) with the energy minimized nsp14 and nsp16 proteins. For this, both proteins were added with polar hydrogens along with Kollman charges and the Lamarckian genetic algorithm was employed with 10 GA (Genetic Algorithm) runs for the docking protocol. The protein nsp14 was subjected to a grid box (15.178 × − 8.265 × − 19.955 Å) with a default spacing of 0.375 Å, which was centered and aligned encompassing the binding pocket of the nsp14 protein. Similarly, for nsp16 protein, grid box was set to 81.790 × 14.047 × 2.616 Å with a default spacing of 0.375 Å covering the binding pocket of the nsp16 protein. Subsequently, by employing the same parameters, the compounds obtained from the virtual screening were subjected to docking with the refined nsp14 and nsp16 proteins. The binding energies and molecular interactions of the compounds with the nsp14 and nsp16 proteins were compared. The non-bonded molecular interactions present in the complex were observed using Discovery Studio Visualizer and 3D protein visuals were generated using Chimera.

The drug-likeness characteristics of the hit compounds were calculated utilizing PaDEL-Descriptors^32^, for which the ‘SMILES’ notations of the hit compounds were created by Discovery Studio Visualizer. The toxicity profile of the hit compounds was determined using ProTox-2 tool^33^. Additionally, the screened compounds were subjected to the PAINS (pan assay interference compounds) filter, which removes false positive compounds by identifying the chemical sub-structures known to interfere with ligand–protein interactions^34^.

### Molecular dynamics (MD) simulations

To understand the protein–ligand interactions and to validate the docking results molecular dynamics simulations were performed using the GROMACS simulation program (version 2020.2)^35^. All the simulations were performed using ‘PARAM Shakti’ HPC (High Performance Computing) GPU compute nodes on 80 cores of two 2.4 GHz Intel Xeon SKL G-6148 processors with NVIDIA V100 graphic cards. For molecular dynamics simulations, the compound-methyltransferase complexes obtained from docking analysis were utilized. The dynamic system was solvated using TIP3P (transferable intermolecular potential with 3 points) water model in a rectangular simulation box and 0.15 M NaCl (Na^+^  = 41 ions and Cl^–^ = 46 ions) was introduced to neutralize the charge of the solvated system using the Monte-Carlo ion placement method. The atomistic simulations were carried out using the CHARMM36m (Chemistry at Harvard Macromolecular Mechanics) force field^36^. The steepest descent integrator was used for energy minimization of all the systems for 5000 steps. The energy reduced systems were then gradually relaxed and brought into equilibrium using the NVT and NPT ensemble (N = number of molecules, V = volume, T = temperature, and P = pressure). The molecular dynamics production run was performed for 300 ns with 1 Barr pressure maintained by Parrinello-Rahman coupling and temperature maintained at 310 K using a V-rescale modified Berendsen thermostat. The root mean square deviation (RMSD), root mean square fluctuation (RMSF), number of hydrogen bonds, radius of gyration (Rg), and solvent accessible surface area (SASA) were used to analyse the trajectories from the simulation studies. Further, time-dependent changes in the protein conformation upon compound binding were analysed for the entire simulation period.

### Gibbs binding free energy calculations

The bound state and entirely unbound states of the protein and compounds were compared in terms of Gibbs free energy using a molecular mechanics-generalized born surface area (MM-GBSA) study. The *gmx_MMPBSA* module via GROMACS was used for the calculations^37^. The Gibbs binding free energy calculations were resolved based on molecular mechanical energy changes in the gas phase (van der Waals energy + electrostatic energy) and free energy of solvation (polar energy + non-polar solvation energy) as shown in Eq. (1) and (2). Finally, the *gmx_MMPBSA_ana* module was utilized to analyse the calculated results.

1 $$\Delta {\text{G}}_{{{\text{bind}}}} = \Delta {\text{E}}_{{{\text{Gas}}}} + \Delta {\text{E}}_{{{\text{Solv}}}}$$

where ΔG_bind_, ΔE_Gas_ and ΔE_Solv_ represent binding free energy, molecular mechanical energy changes in the gas phase and energy of solvation respectively.

2 $$\Delta {\text{G}}_{{{\text{bind}}}} = \, \left( {\Delta {\text{E}}_{{{\text{vdW}}}} + \, \Delta {\text{E}}_{{{\text{ele}}}} } \right) \, + \, \left( {\Delta {\text{E}}_{{{\text{polar}}}} + \, \Delta {\text{E}}_{{{\text{non}} - {\text{polar}}}} } \right)$$

where ΔE_vdW_, ΔE_ele_, ΔE_polar_ and ΔE_non-polar_ depict van der Waals energy, electrostatic energy, polar solvation energy and non-polar solvation energy respectively.

### Density functional theory (DFT) calculations

DFT computer modeling was used to examine the electronic, atomic, and energetic states of the hit compounds, as described earlier^38^. Prior to the DFT calculations, the GaussView 6.1.1 software was utilized to prepare and energetically optimize the hit compounds^39^. The calculations were carried out using the B3LYP (Becke’s 3-parameter Lee–Yang–Parr) method with 6–31 +  + G (d, p) basis set for the atoms of carbon, oxygen, nitrogen, sulphur, and hydrogen in the Gaussian 16W software [46]. Total energies, dipole moment, HOMO (highest occupied molecular orbital) energy, LUMO (lowest unoccupied molecular orbital) energy, and band gap energy (ΔG = E_LUMO_ − E_HOMO_) were calculated for the hit compounds. Frontier molecular orbitals (FMO) and molecular electrostatic potential (MEP) investigations were also carried out to predict the chemical reactivity and reactive sites of the hit compounds.

## Results and discussion

### Virtual screening of AGP derivatives

Amongst 413 compounds, the top 10 compounds having the least binding energy were selected (Table S1 and S2 in Supplementary Information). In the case of nsp14 protein, the binding energy was observed from − 11.40 (44437491) to − 10.30 kcal/mol (138968421), whereas in the case of nsp16, BE range was − 8.40 kcal/mol (138968421) to − 8.20 kcal/mol (132210553). Further, these top ten compounds were observed to have better binding energy than the co-crystal SAH (− 7.42 kcal/mol) and co-crystal SFG (− 7.49 kcal/mol). The goal of the study is to find a common drug-like candidate that can inhibit both nsp14 and nsp16 proteins, the binding energies of all the screened andrographolide derivatives against both the proteins were compared. Among the top 10 screened compounds, four compounds, i.e. 44437487, 44437491, 2734589 and 138968421, were found to be common.

### Molecular docking of the hit compounds with nsp14 and nsp16 proteins

The drug-likeness properties of the compounds were assessed by employing PaDEL-descriptors and the ProTox-2 tool. In order to be considered a drug-like candidate, a molecule generally needs to meet all the criteria outlined in Lipinski's Rule of Five. These criteria include a molecular weight of less than 500 g/mol, fewer than 10 hydrogen bond acceptors, fewer than 5 hydrogen bond donors, and the MLogP (Moriguchi LogP) value of less than 4.15. Out of four compounds, only two (2734589, 138968421) qualified the Lipinski’s Rule of Five, whereas the other two compounds (44437487, 44437491) showed slightly higher values for molecular weight and MLogP (Table 1). However, we have considered all four compounds for further investigation as for respiratory diseases, inhalation route (spray/aerosol) is also preferred for which Lipinski’s Rule of Five need not to be qualified^40^.

**Table 1:** Comparison of drug-likeness properties and toxicity profiling of the hit compounds predicted using PaDEL-Descriptors application.

Parameters	44437487	44437491	2734589	138968421	SAH	SFG
Binding energy (nsp14/nsp16) (kcal/mol)	− 12.60/ − 8.00	− 12.15/ − 9.74	− 11.10/ − 8.45	− 10.00/ − 8.76	− 7.42	− 7.49
Molecular weight (g/mol)	676.84	673.80	466.53	448.59	381.39	384.41
H-bond acceptors	8	9	6	6	10	11
H-bond donors	0	0	2	1	6	5
LogP	5.03	4.28	2.80	3.26	− 4.32	− 4.63
Lipinski rule violations	2	2	0	0	1	1
Toxicity class	5	5	4	4	5	4
Estimated LD_50_ (mg/kg)	5000	5000	700	500	3320	1000

The examination of toxicity predicted that compounds 44437487 and 44437491 have a class five toxicity with comparatively higher estimated LD_50_ value (5000 mg/kg) with a prediction accuracy of approximately 67%. On the other hand, compounds 2734589 and 138968421 exhibited a class four toxicity, as evidenced by their anticipated LD_50_ values of 700 mg/kg and 500 mg/kg respectively with a prediction accuracy of 67%. Concurrently, the analysis of cytotoxicity, mutagenicity, hepatotoxicity, and carcinogenicity activities revealed that all the compounds exhibit an inactive state with a score ranging between 0.50 and 0.99. This clearly demonstrates that the selected four compounds have no toxicological effects and thereby can be used as drug molecules. Moreover, all four compounds successfully passed the PAINS filter showing the absence of chemical sub-structures in the compounds that could interfere in biological studies.

**Figure 1:**
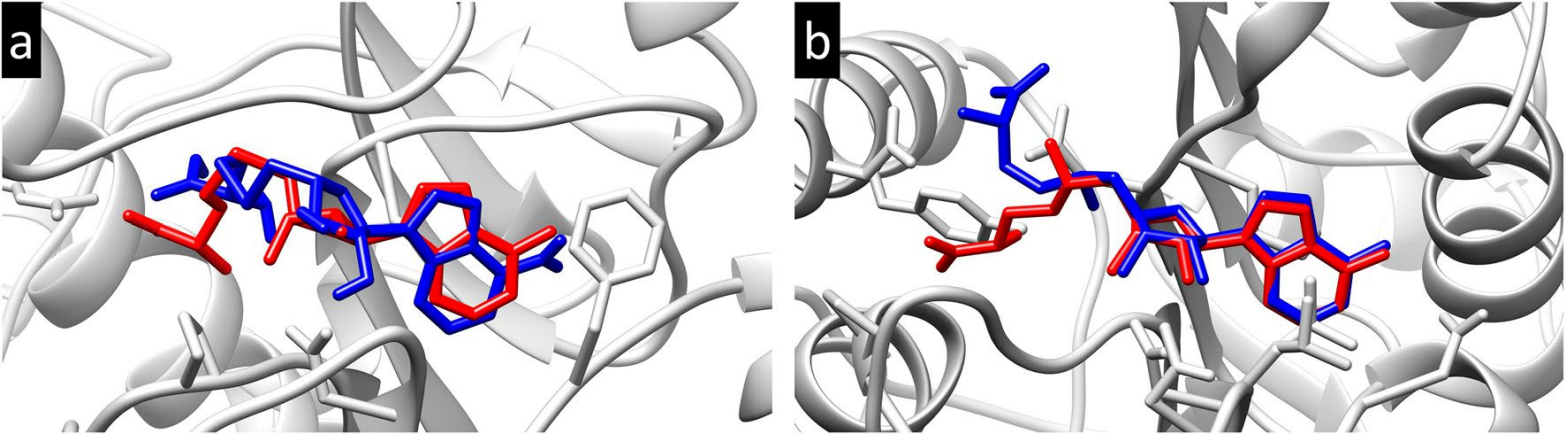
3D illustration of the superimposed (**a**) SAH and (**b**) SFG at the active site of nsp14 and nsp16 protein respectively. Color code: co-crystal SAH/SFG (red) and re-docked SAH/SFG (blue).

Prior to performing molecular docking, the docking process was validated by re-docking the SAH/SFG to the binding pocket of the nsp14/nsp16 protein. Figure 1 shows the superimposed structure of the co-crystal SAH/SFG with the docked SAH/SFG indicating the occupancy at the same binding pocket with an RMSD value of 0.40 Å and 0.44 Å respectively for nsp14 and nsp16 complexes. Therefore, the same docking parameters were employed to investigate the molecular level interactions of the common compounds with the protein targets.

**Figure 2:**
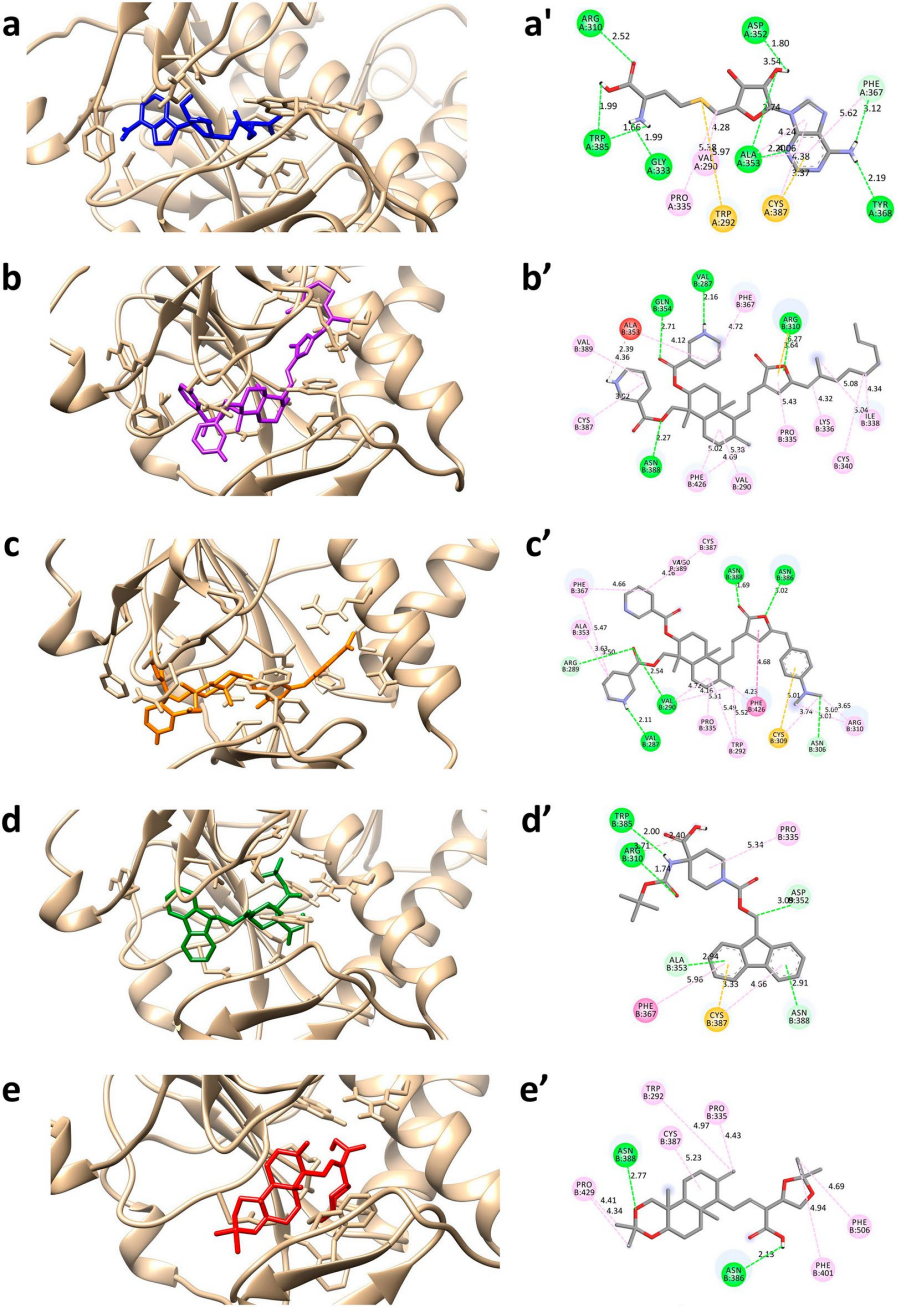
The molecular interactions between the hit compounds and the nsp14 protein with 3D (left panel, **a**→**e**) and 2D (right panel, **a’**→**e’**) representations. The protein structure is represented in the tan color and the compounds are depicted with stick models; (**a**,**a’**) SAH, (**b**,**b’**) 44437487, (**c**,**c’**) 44437491, (**d**,**d’**) 2734589 and (**e**,**e’**) 138968421.

In order to get insight into the interactions occurring between the hit compounds and the protein targets, 2D and 3D molecular interactions were investigated. The analysis revealed that the stabilizing forces between the hit compounds and the protein are largely hydrogen bonds and hydrophobic interactions (Tables 2, 3). The docked poses of all the compounds with nsp14 protein exhibited a common binding pocket (Fig. 2). The 2D images of the compounds with atom numbering are provided in Figure S1 (Supplementary Information). The binding interaction analysis of SAH with nsp14 revealed the formation of 9 hydrogen bonds (Fig. 2a and Table 2). The oxygen atom at 26th position formed a hydrogen bond with Arg310 (bond length (BL) of 2.52 Å) whereas the Trp385 formed two conventional hydrogen bonds with hydroxyl (BL = 1.99 Å) and amino group (BL = 1.66 Å) at 23rd and 25th position respectively. The amino group at 25th position also participated in hydrogen bonding with Gly333 (BL = 1.99 Å), while the Ala353 residue formed hydrogen bond with hydroxyl group at 15th position and imidazole ring of adenine moiety with bond lengths of 2.74 Å and 2.20 Å respectively. The hydroxyl group at 15th position also contributed to hydrogen bonding with Asp352 (BL = 1.80 Å). The amino group on the imidazole group formed a hydrogen bond with Tyr368 (BL = 2.19 Å). Carbon hydrogen bond and π-donor hydrogen bond were also formed by Asp352 and Cys367 with furan ring (BL = 3.54 Å) and amino group on imidazole moiety (BL = 3.12 Å) respectively. Apart from this, Cys387 formed two hydrophobic interactions with adenine moiety with bond lengths 3.37 Å and 4.38 Å, whereas the sulphur atom at the 18th position formed hydrophobic interaction with Trp292 (BL = 5.97 Å). The hydrogen atom at 17th position formed hydrophobic interactions with both Val290 and Pro335 with bond lengths 4.28 Å and 5.38 Å respectively. The residue Cys353 formed two hydrophobic interactions with adenine moiety of bond lengths of 4.06 Å and 4.24 Å. Finally, Phe367 formed hydrophobic interactions with imidazole group on the adenine moiety with a bond length of 5.62 Å (Table 2 and Fig. 2a).

**Table 2:** Overview of types of molecular interactions between the hit compounds and the nsp14 protein.

Compound	Interacting residues (bond distance in Å)		
	Hydrogen bonds	Hydrophobic interactions	Electrostatic interactions
SAH	Asp352:OD2 (3.54), Phe367 (3.12) Trp385:O (1.66), Gly333:O (1.99), Asp352:OD1 (1.80), Trp385:O (1.99), Ala353:HN (2.74), Tyr368:O (2.19), Arg310:HH22 (2.52), Ala353:HN (2.20),	Cys387 (4.38), Ala353 (4.06) Pro335 (5.38), Ala353 (4.24), Phe367 (5.62), Val290 (4.28), Cys387 (3.37), Trp292 (5.97),	Nil
44437487	Asn388:HN (2.27), Val287:O (2.16) Arg310:HH12 (2.24), Gln354:HN (2.71),	Phe426 (4.69), Pro335 (5.43) Phe367 (4.72), Phe426 (5.02), IlE338 (4.34), VaL389 (4.36), Cys387 (3.92), IlE338 (5.08), CyS340 (5.04), AlA353 (4.12), Val290 (5.38), LyS336 (4.32),	Arg310:NH2 (3.64)
44437491	Arg289:CA (3.50), Asn306:O (3.01) Asn388:HD21 (1.69), Val287:O (2.11), Val290:HN (2.54), Asn386:HD22 (3.02),	Phe367 (4.66), Phe426 (4.23) Trp292 (5.49), Phe367 (5.47), Val389 (4.16), Trp292 (5.52), Cys309 (3.74), Arg310 (3.65), Val290 (4.16), Arg310 (5.09), Ala353 (3.63), Cys387 (4.30), Val290 (4.72), Pro335 (5.31), Cys309 (5.01), Phe426 (4.68),	Nil
2734589	Asn388:HN (2.91) Asp352:OD2 (3.09), Ala353:HN (2.94), Arg310:HH22 (1.74), Trp385:O (2.00),	Pro335 (5.34), Cys387 (4.66) Cys387 (3.33), Phe367 (5.96),	Nil
138968421	Asn388:HD22 (2.77), Asn386:OD1 (2.13)	Phe506 (4.69) Trp292 (4.97), Phe401 (4.94), Pro429 (4.34), Pro429 (4.41), Cys387 (5.23), Pro335 (4.43),	Nil

**Table 3:** Overview of types of molecular interactions between the hit compounds and the nsp16 protein.

Compound	Interacting residues (bond distance in Å)		
	Hydrogen bonds	Hydrophobic interactions	Electrostatic interactions
SFG	Gly6911:O (3.32) Cys6913:SG (3.44), Tyr6930:O (2.87), Asp6928:OD2 (2.71), Asp6912:OD1 (2.60), Gly6871:O (2.94), Asp6897:OD2 (3.08), Lys6968:HZ1 (1.74), Gly6869:O (2.74), Asn6899:HD22 (2.05), Cys6913:HN (1.99), Leu6898:HN (2.18), Asn6899:HD21 (3.10),	Cys6913 (4.83) Leu6898 (4.81), Met6929 (4.79), Leu6898 (3.87), Met6929 (3.69),	Lys6968:NZ (2.86) Lys6844:HZ2 (6.14),
44437487	Asp6928:OD2 (3.16) Asp6928:O (2.23), Asn6899:OD1 (2.22), Leu6898:HN (2.79), Cys6913:HN (2.23),	Cys691 3 (4.76) Phe6947 (5.48), Leu6898 (4.72), Leu6898 (4.87), Pro6932 (5.31), Pro6932 (4.41), Met6929 (4.99), Met6929 (3.67), Cys6913 (5.21),	Nil
44437491	Tyr6930:HN (2.48) Gly6869:CA (3.17), Gly6869:O (3.08), Asn6841:HD21 (2.32), Gly6871:O (1.88),	Leu6898 (4.71), Met6929 (5.42) Met6929 (5.07), Leu6898 (4.64),	Asp6912:OD1 (3.79) Asp6897:OD2 (3.25),
2734589	Gly6869:O (3.43), Tyr6930:O (2.59) Asp6928:OD2 (2.95), Gly6871:O (3.39), Tyr6845:HH (2.88), Gly6871:O (2.17),	Leu6898 (4.62), Cys6913 (4.87) Pro6878 (4.74), Tyr6845 (4.97), Leu6898 (3.72), Met6929 (3.55),	Nil
138968421	Asp6928:OD2 (3.27), Asp6897:OD2 (3.25) Leu6898:HN (2.85), Lys6968:HZ1 (1.59), Asn6841:HD21 (2.07), Lys6844:HZ3 (2.12),	Leu6898 (3.90), Met6929 (3.79), Phe6947 (4.62)	Nil

The compound 44437487 formed four conventional hydrogen bonding with the nsp14 protein (Fig. 2b and Table 2). The oxygen atom at 23rd position on the furan ring formed a hydrogen bond with Arg310 with a length of 6.27 Å. The oxygen atom on the 8th position participated in hydrogen bonding with Asn388 (BL = 2.27 Å), whereas oxygen atom on 38th position formed a conventional hydrogen bond with Gln354 (BL = 2.71 Å). Similarly, the nitrogen atom at 47th position formed hydrogen bonding with Val287 with bond length of 2.16 Å. A single electrostatic interaction was formed by furan moiety of the compound and Arg310 with a bond length of 3.64 Å. Apart from these, 12 hydrophobic interactions were formed among the protein and 44437487. The methyl groups at the aliphatic chain formed four hydrophobic interactions with Lys336 (BL = 4.32 Å), Cys340 (BL = 5.04 Å) and Ile338 (BL = 5.08 Å and 4.32 Å) whereas the connected furan ring formed interaction with Pro335 (BL = 5.43 Å). The cyclohexane and methyl group at 49th position formed three hydrophobic interactions with Phe426 (BL = 5.02 Å and 4.69 Å) and Val290 (BL = 5.38 Å). The 1st pyridine moiety participated in hydrophobic interactions with Cys387 (BL = 3.92 Å) and Val389 (BL = 4.36 Å) whereas the 2nd pyridine moiety formed interactions with Ala353 (BL = 4.12 Å) and Phe367 (BL = 4.72 Å). Similarly, in compound 44437491, the oxygen atom on furan ring and oxygen atom at 47th position formed conventional hydrogen bonds with Asn386 (BL = 3.02 Å) and Asn388 (BL = 1.69 Å) respectively. Likewise, oxygen atom at 23rd position formed hydrogen bond with Val290 with a bond length of 2.54 Å. The nitrogen atom at 27th position formed hydrogen bond with Val287 with a bond length of 2.11 Å. Apart from this, two carbon hydrogen bonds were formed between Arg289 and Asn306 with oxygen atom at 51st position and methyl group at 45th position respectively. The complex was also stabilized by forming 17 hydrophobic interactions. The methyl group at 29th position formed three hydrophobic interactions with Phe426 (BL = 4.23 Å), Trp292 (BL = 5.52 Å) and Val290 (BL = 4.16 Å), whereas cyclohexane at 16th position formed three hydrophobic interactions with Val290 (BL = 4.72 Å), Pro335 (BL = 5.31 Å) and Trp292 (BL = 5.49 Å). The methyl group at 45th and 46th positions formed three hydrophobic interactions with Arg310 (BL = 3.65 Å and 5.09 Å) and Cys309 (BL = 3.74 Å). The benzene ring formed a hydrophobic interaction with Cys309 with a bond length of 5.01 Å. The pyridine moiety at 1st position formed three hydrophobic interactions with Phe367 (BL = 4.66 Å), Val389 (BL = 4.16 Å) and Cys387 (BL = 4.30 Å). Likewise, the pyridine moiety at 22nd position also formed two hydrophobic interactions with Phe367 and Ala353 with bond lengths of 5.47 Å and 3.63 Å respectively. Finally, a hydrophobic interaction was formed between Phe426 and furan moiety with a bond length of 4.68 Å (Table 2 and Fig. 2c).

In the case of compound 2734589, two conventional hydrogen bonds were formed along with a carbon hydrogen bond and two π-donor hydrogen bonds. The oxygen atom at 33rd position formed hydrogen bond with Arg310 (BL = 1.74 Å) and amine group at 26th position participated in hydrogen bonding with Trp385 (BL = 2.00 Å). Asp352 formed carbon hydrogen bonding with methyl group at 14th position (BL = 3.09 Å). The π-donor hydrogen bonds were formed between the benzene rings and Asn388 (BL = 2.91 Å) and Ala353 (BL = 2.94 Å). The benzene rings also contributed to hydrophobic interaction with Cys387 (BL = 3.33 Å and 4.66 Å) and Phe367 (BL = 5.96 Å). Finally, Pro335 formed interactions with cyclohexane moiety at 20th position with bond length of 5.34 Å (Table 2 and Fig. 2d). In case of the compound 138968421, Asn388 and Asn386 formed conventional hydrogen bonds with oxygen atom on 1st position (BL = 2.77 Å) and hydroxyl group at 20th position (BL = 2.13 Å) respectively. Apart from this, seven hydrophobic interactions were formed in the complex. The methyl group at 26th position formed two hydrophobic interactions with Phe506 and Phe401 with bond lengths of 4.69 Å and 4.94 Å respectively. The oxygen atom at 28th position participated in hydrophobic interactions with Trp292 (BL = 4.97 Å) and Pro335 (BL = 4.43 Å). The Pro429 formed two interactions with methyl groups at 29th and 30th position on the compound with bond lengths of 4.34 Å and 4.41 Å respectively. Finally, the cyclohexane moiety formed a hydrophobic interaction with Cys387 with a length of 5.23 Å (Table 2 and Fig. 2e).

**Figure 3:**
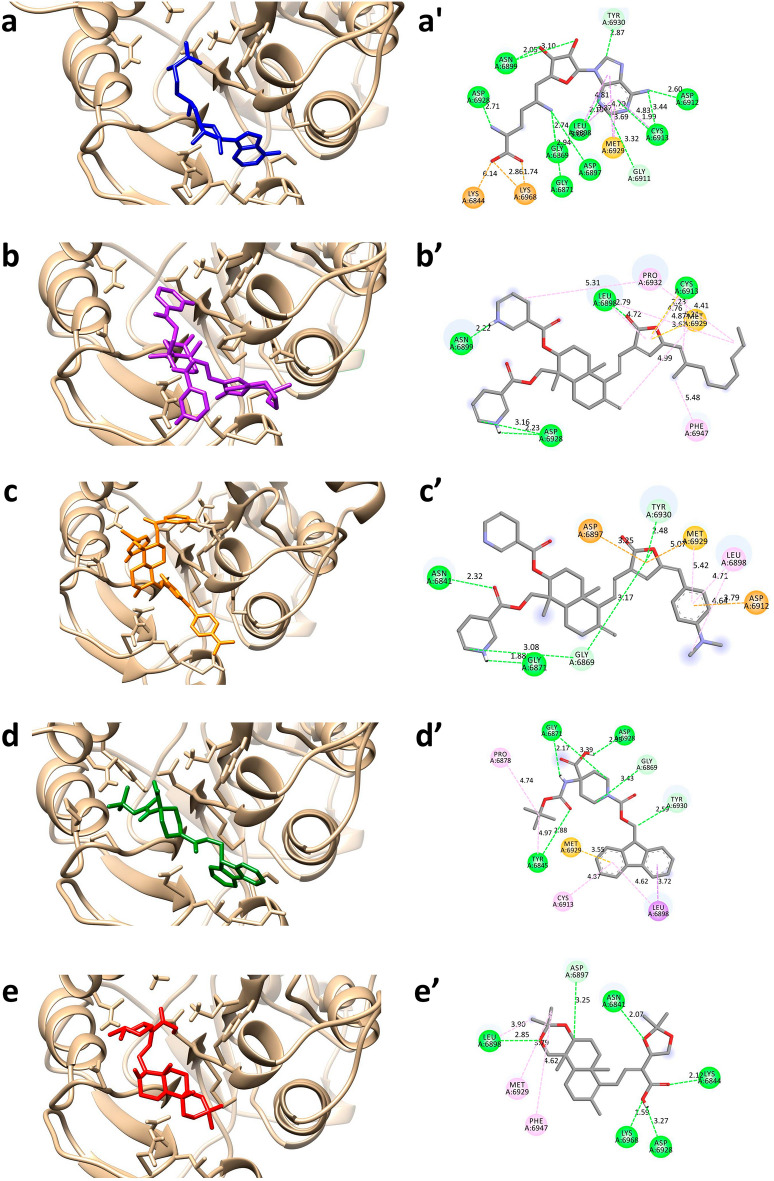
The molecular interactions between the hit compounds and the nsp16 protein with 3D (left panel, **a**→**e**) and 2D (right panel, **a’**→**e’**) representations. The protein structure is represented in the tan color and the compounds are depicted with stick models; (**a**,**a’**) SFG, (**b**,**b’**) 44437487, (**c**,**c’**) 44437491, (**d**,**d’**) 2734589 and (**e**,**e’**) 138968421.

The docked poses and binding interactions of the hit compounds with nsp16 protein are shown in Fig. 3. The binding interaction analysis of SFG with nsp16 revealed 13 hydrogen bonds with the nsp16 protein. The imidazole group on adenine moiety formed hydrogen bonds with Asp6912 (BL = 2.60 Å), Cys6913 (BL = 1.99 Å and 3.44 Å) and Leu6898 (BL = 2.18 Å). The amine group at 27th position participated in conventional hydrogen bonding with Asp6897 (BL = 3.08 Å), Gly6869 (BL = 2.74 Å) and Gly6871 (BL = 2.94 Å). The hydroxyl group at 15th and 16th position formed interactions with Asn6899 with bond lengths 2.05 Å and 3.10 Å respectively. The oxygen atom at 26th position and amino group at 25th position formed hydrogen bond with Lys6968 (BL = 1.74 Å) and Asp6928 (BL = 2.71 Å) respectively. The adenine moiety also participated in carbon hydrogen bond formation with Tyr6930 (BL = 2.87 Å) and Gly6911 (BL = 3.32 Å). The hydroxyl group at 23rd position and oxygen atom at 26th position formed electrostatic interactions with Lys6844 (BL = 6.14 Å) and Lys6968 (BL = 2.86 Å). Apart from this, the adenine moiety formed hydrophobic interactions with Leu6898 (BL = 3.87 Å), Met6929 (BL = 3.69 Å), Leu6898 (BL = 4.81 Å), Met6929 (BL = 4.79 Å) and Cys6913 (BL = 4.83 Å) (Table 3 and Fig. 3a). In case of compound 44437487, oxygen atoms at 23rd and 26th positions formed a conventional hydrogen bond with Cys6913 (BL = 2.23 Å) and Leu6898 (BL = 2.79 Å) respectively. The nitrogen atoms at 3rd and 47th positions formed hydrogen bonds with Asp6928 (BL = 2.23 Å and 3.16 Å) and Asn6899 (BL = 2.22 Å) respectively. Apart from these, nine hydrophobic interactions were formed between compound and Met6929 (BL = 3.67 Å), Cys6913 (BL = 5.21 Å), Pro6932 (BL = 4.41 Å), Met6929 (BL = 4.99 Å), Leu6898 (BL = 4.87 Å), Pro6932 (BL = 5.31 Å), Phe6947 (BL = 5.48 Å), Leu6898 (BL = 4.72 Å) and Cys6913 (BL = 4.76 Å) (Table 3 and Fig. 3b).

In the compound 44437491, the oxygen atom at 23rd position formed a hydrogen bond with Asn6841 with a bond length of 2.32 Å. The nitrogen atom at 27th position formed another hydrogen bonding with Gly6871 with a bond length of 1.88 Å. Apart from this, two carbon hydrogen bonds were formed between Gly6869 and carbon atom at 26th position and furan moiety with bond lengths of 3.08 Å and 3.17 Å respectively. The furan moiety also participated in π-donor hydrogen bond with Tyr6930 with a bond length of 2.48 Å. In addition, four hydrophobic interactions were formed within the complex. The residue Met6929 formed a hydrophobic interaction with furan moiety with a bond length of 5.07 Å. The benzene moiety at 38th position and methyl group at 46th position formed hydrophobic interactions with Leu6898 (BL = 4.71 Å and 4.64 Å) and Met6929 (BL = 5.07 Å) respectively. The presence of electrostatic interactions was also observed within the complex. The furan moiety and benzene moiety participated in electrostatic interactions with Asp6897 (BL = 3.25 Å) and Asp6912 (BL = 3.79 Å) respectively (Table 3 and Fig. 3c).

The compound 2734589 formed three conventional hydrogen bonds; the oxygen atom at 30th position, hydroxyl group at 24th position and amino group at 26th position with Tyr6845 (BL = 2.88 Å), Asp6928 (BL = 2.95 Å) and Gly6871 (BL = 2.17 Å) respectively. The cyclohexane moiety at 20th position and methyl group at 14th position formed three carbon hydrogen bonds with Gly6871 (BL = 3.39 Å), Gly6869 (BL = 3.43 Å) and Tyr6930 (BL = 2.59 Å) respectively. The two benzene rings formed hydrophobic interactions with Leu6898 (BL = 3.72 Å and 4.62 Å), Cys6913 (BL = 4.87 Å) and Met6929 (BL = 3.55 Å). Finally, carbon atom at 29th position formed two hydrophobic interactions with Tyr6845 (BL = 4.97 Å) and Pro6878 (BL = 4.74 Å) (Table 3 and Fig. 3d). In case of compound 138,968,421, the oxygen atom at 25th and 19th position formed hydrogen bonding with Asn6841 (BL = 2.07 Å) and Lys6844 (BL = 2.12 Å) respectively. The hydroxyl group at 20th position formed two hydrogen bonds with Lys6968 (BL = 1.59 Å) and Asp6928 (BL = 3.27 Å). The oxygen atom at 3rd position formed a conventional hydrogen bonding with Leu6898 (BL = 2.85 Å). Asp6897 formed a carbon hydrogen bond with hydrogen atom at 32nd position (BL = 3.25 Å). In addition, hydrophobic interactions were observed between methyl groups at 29th and 30th positions and Leu6898 (BL = 3.90 Å), Met6929 (BL = 3.79 Å) and Phe6947 (BL = 4.62 Å) (Table 3 and Fig. 3e).

Upon conducting a more detailed analysis of the molecular docking interactions, it was observed that all common four hit compounds exhibited a comparatively lower number of hydrogen bonds in comparison to co-crystal compounds (SAH/SFG). However, the energy contribution (or binding affinity) by hydrophobic and electrostatic interactions is observed to be greater compared to hydrogen bonds and thus the overall binding energy is lesser for them than the SAH/SFG. This observation indicates a higher binding preference of the hit compound towards the nsp14/nsp16 protein in comparison to SAH/SFG. For further validation, the best binding conformations (orientation) of the hit compounds with the nsp14 and nsp16 targets were selected.

### Molecular dynamics simulations

**Figure 4:**
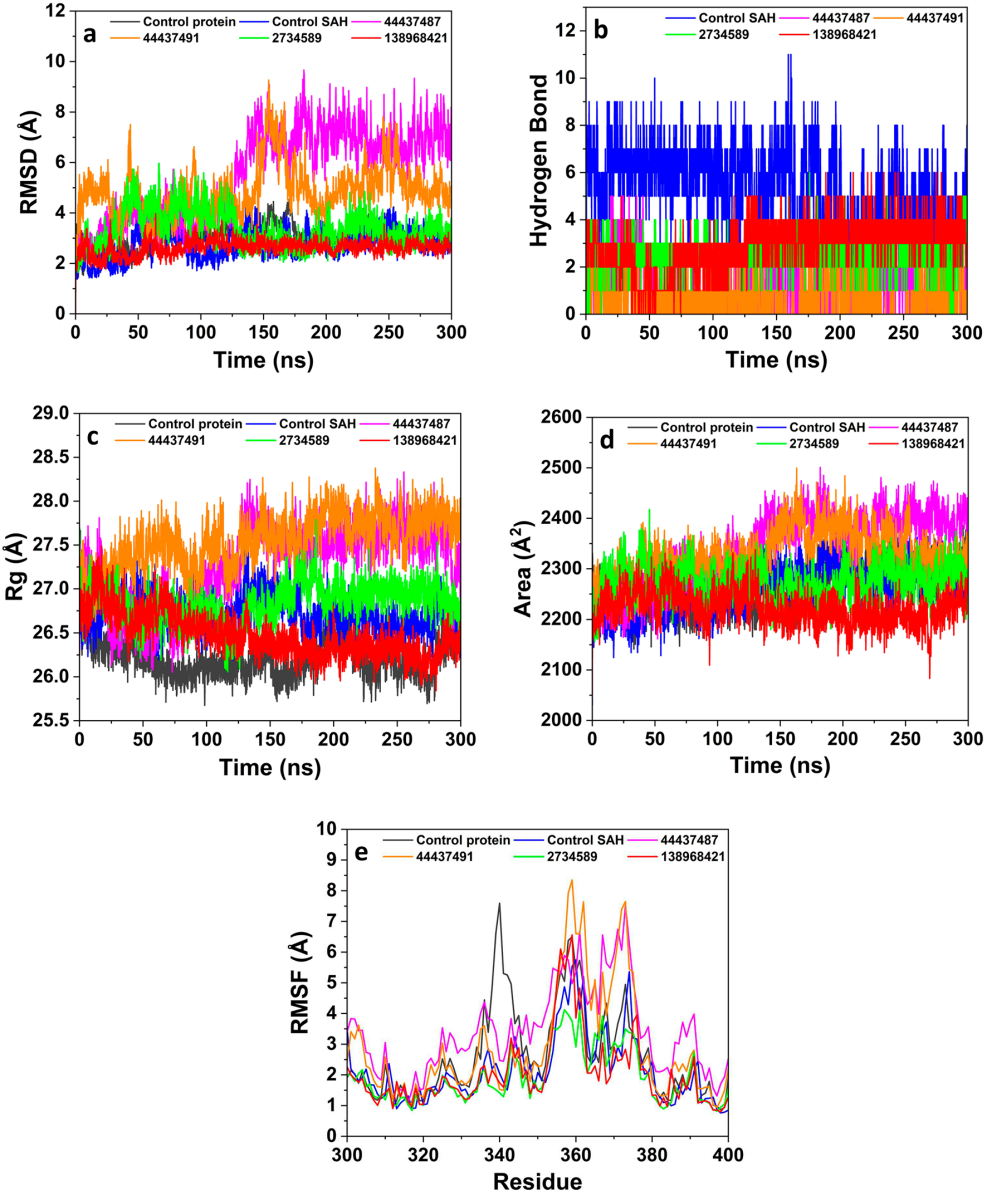
Comparison of molecular dynamics simulations analysis of the hit compounds and control complexed with nsp14 protein during a 300 ns simulation period; (**a**) RMSD, (**b**) hydrogen bond formations, (**c**) radius of gyration (Rg), (**d**) solvent accessible surface area (SASA), and (**e**) RMSF plot.

To establish the stability of the MTs (nsp14 and nsp16) and hit compound complexes in the biological system, molecular dynamics simulations were performed. The dynamic trajectory analysis revealed that all four hit compounds have better or equivalent stability and rigid dynamic behavior as compared to respective control co-crystal molecules complexed with the MTs. The compounds 138968421 and 2734589 showed lower oscillations in the MD trajectory compared to control protein, SAH and compounds 44437487 and 44437491, suggesting their higher stability at the binding pocket of the nsp14 protein (Fig. 4a). The compounds 138968421 and 2734589 having the average RMSD values of 2.6 Å and 3.2 Å, respectively, exhibited a consistent signal throughout the simulation trajectory, which suggests their more stable and rigid confirmation by virtue of their strong and stable interactions with the nsp14 protein. On the other hand, the compounds 44437487 and 44437491 showed prominent fluctuations throughout the simulation trajectory, and the overall average RMSD value was observed to be 5.5 Å and 4.8 Å respectively which were higher than the control protein (3.0 Å) and control SAH (2.7 Å). Further, the protein and its complex with SAH showed oscillations comparable to the compounds 138968421 and 2734589.

The RMSF analysis corroborated the outcome of RMSD analysis on the magnitude of fluctuations. The hit compounds showed an average RMSF value in the range of 1.9 Å to 3.4 Å with control protein and control SAH showing an average value of 2.6 Å and 2.1 Å respectively (Fig. 4e). The compounds 138968421 (2.0 Å) and 2734589 (1.9 Å) exhibited the most stable nature in the binding pocket of the protein showing a lower magnitude of fluctuations. The compounds 44437487 and 44437491 exhibited the most significant variations in the binding pocket of the protein across the simulation duration, as indicated by an average RMSF value of 3.4 Å and 2.9 Å respectively. Further, the hydrogen bonds analysis revealed that the hit compounds exhibit a lesser number of hydrogen bonds (1–3 H bonds) in comparison to the control SAH (~ 5 H bonds) (Fig. 4b), which is in good agreement with the docking analysis. The compounds 138968421 and 2734589 exhibited an average number of ~ 3 hydrogen bonds, while the other two compounds formed an average of ~ 1 hydrogen bond throughout the simulation period. Although a relatively reduced occurrence of hydrogen bond formation is observed for the hit compounds in comparison to SAH, the stability of these hit compounds with nsp14 protein is mostly sustained by hydrophobic and/or electrostatic interactions as evidenced by docking results.

The Rg analysis revealed an average score of 27.27 Å (44437487), 27.55 Å (44437491), 26.83 Å (2734589) and 26.47 Å (138968421), whereas the control protein and control SAH showed an average Rg score of 26.21 Å and 26.71 Å respectively (Fig. 4c). However, significant variations in the Rg score were noted for the compounds 44437487 and 44437491, whereas the other compounds and the controls showed fluctuations in a very narrow range. This infers that the protein undergoes suitable folding to achieve a desirable level of compactness while maintaining its conformational shape over the whole simulation period following its interaction with the hit compounds. The curve representing the SASA exhibited a consistent trend for all the compounds, as well as the controls. The mean surface area values for the protein, SAH, 44437487, 44437491, 2734589 and 138968421 were determined to be 2261.29 Å^2^, 2340.80 Å^2^, 2330.40 Å^2^, 2273.58 Å^2^ and 2222.19 Å^2^ respectively (Fig. 4d). This suggests that there is no shifting of amino acid residues from the solvent accessible area in the protein during the simulation period. Additionally, even after the protein binds with the compound, its conformational structure and compactness remain unchanged.

**Figure 5:**
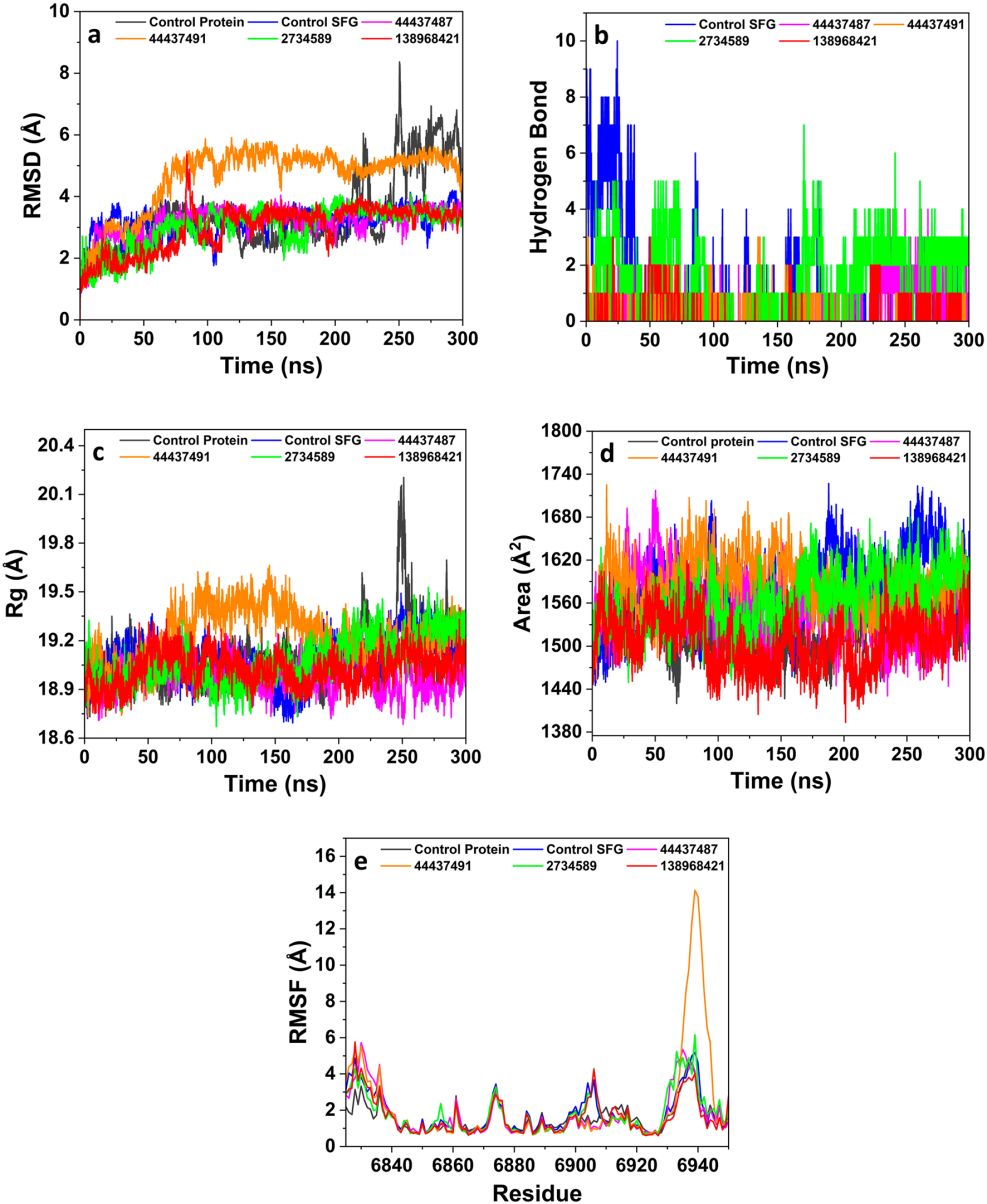
Comparison of molecular dynamics simulations analysis of the hit compounds and controls with nsp16 protein during a 300 ns simulation period; (**a**) RMSD, (**b**) hydrogen bond formations, (**c**) radius of gyration (Rg), (**d**) solvent accessible surface area (SASA), (**e**) RMSF plot.

In the case of nsp16 protein, the RMSD curve of the compounds 44437487, 2734589, 138968421 and control SFG exhibited lesser oscillations as compared to control protein, and compound 44437491, which suggests their stable behavior at the binding pocket of the protein (Fig. 5a). The compound 44437491 exhibited a higher level of fluctuations throughout the simulation period with an average RMSD value of 4.6 Å. Similarly, the control protein exhibited significant oscillations after 200 ns with an average RMSD value of 3.4 Å. The compounds 44437487 (3.1 Å), 2734589 (3.0 Å) and 138968421 (2.9 Å) showed relatively lesser RMSD deviations compared to 44437491 and controls. This implies that these three compounds have stable interactions with the nsp16 protein. Further, the compounds 2734589 and 138968421 consistently displayed lower fluctuations like SFG throughout the whole simulation trajectory in contrast to the control protein. This suggests that the hit compounds exhibit a higher degree of stability and rigidity in their conformation, which might be attributed to their prominent interactions with the nsp16 protein.

The results obtained from the RMSF corroborate the analysis of RMSD on the extent of fluctuations. The hit compounds and control SFG exhibited an average RMSF in the range of 1.6 Å to 2.2 Å, suggesting a robust and sustained engagement of the compounds within the binding pocket (Fig. 5e). Amongst all the compounds, 44437491 exhibited the most notable fluctuations in the binding pocket of the protein, with an average RMSF value of 2.2 Å, whereas the compound 138968421 showed the least fluctuations (1.6 Å). In contrast, the control protein showed a slightly lower RMSD value (1.6 Å) than control SFG (1.8 Å) and other test compounds except for 138968421. The investigation of hydrogen bonding formation revealed that compound 2734589 forms an average hydrogen bond count of ~ 2, whereas the remaining three compounds had an average of ~ 1 hydrogen bond during the simulation period. Comparatively, control SFG formed only ~ 1 H bonds throughout the simulation period, which is not in agreement with molecular docking analysis. This might be ascribed to lesser stability and poor interaction of the SFG at binding pocket and thereby it comes out of pocket during the simulations (Fig. 5b).

The Rg measurements indicated the average scores of 18.98 Å (44437487), 19.22 Å (44437491), 19.08 Å (2734589) and 19.02 Å (138968421) which is comparable to the control protein (19.09 Å) and control SFG (19.08 Å) (Fig. 5c). Overall, the controls and the hit compounds demonstrated Rg score variability in a very narrow range suggesting appropriate folding, attainment of a suitable compactness and preservation of the conformational shape of the protein upon interaction with the hit compounds. The trend seen in the SASA curve remained constant for all the compounds, including the controls. The average SASA values for the protein, SFG, 44437487, 44437491 2734589 and 138968421 were determined to be 1525.33 Å^2^, 1582.37 Å^2^, 1550.06 Å^2^, 1585.61 Å^2^, and 1563.22 Å^2^ and 1507.20 Å^2^ respectively (Fig. 5d). This observation implies that there is no movement of amino acid residues from the solvent accessible region within the protein throughout the duration of the simulation. Furthermore, subsequent to the binding of the protein with the compound, the conformational structure and compactness of the protein remain unaltered.

**Figure 6:**
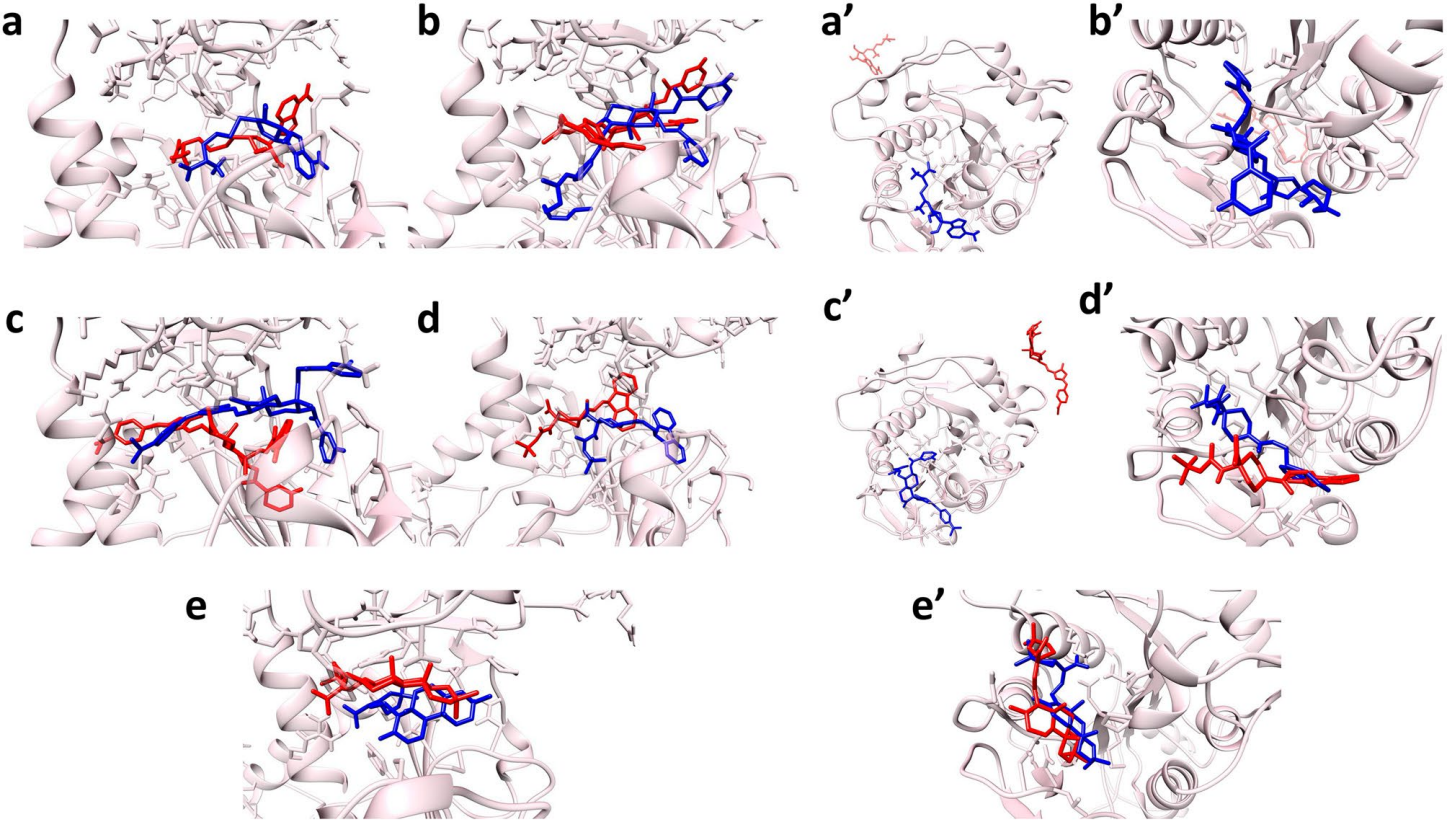
Spatial shifting of the compounds from the binding pocket of the nsp14 (left) and nsp16 (right) protein showing initial (blue colour) and final (red colour) conformation of (**a**,**a’**) SAH, (**b**,**b’**) 44437487, (**c**,**c’**) 44437491, (**d**,**d’**) 2734589 and (**e**,**e’**) 138968421.

Further, to investigate the dynamic changes in protein and characteristics of the compounds, the initial conformations versus final conformations were explored**.** The snapshots for initial and final time points were retrieved from the 300 ns MD trajectory file (.xtc file). In the context of nsp14 protein complexes, it was noted that all the hit compounds remained within the binding pocket of the protein for the entire simulation period with a very minute conformational alteration (Fig. 6). In the case of compounds 44437491 and 2734589, minor modifications were observed in the binding position, which might be attributed to the decreased stability of the compounds within the binding pocket. In the case of nsp16 protein complexes, the compounds 2734589 and 138968421 showed appropriate binding in the binding pocket of the protein throughout the simulation period (Fig. 6). Even though there are some fluctuations in compound 44437491, it stayed at the binding pocket of the nsp16 protein. On the other hand, the control SFG showed higher conformational changes in the binding pocket of the protein along with the compounds 44437487 and 44437491 which infers that these compounds did not stay at the binding pocket of the nsp16 protein. This suggests the poor stability of the SFG and compounds 44437487 and 44437491 towards the nsp16 protein. Overall, the compounds 2734589 and 138968421 showed stable binding with both nsp14 and nsp16 protein targets with better preservation of their structure.

### Binding free energy calculations MM-GBSA

**Figure 7:**
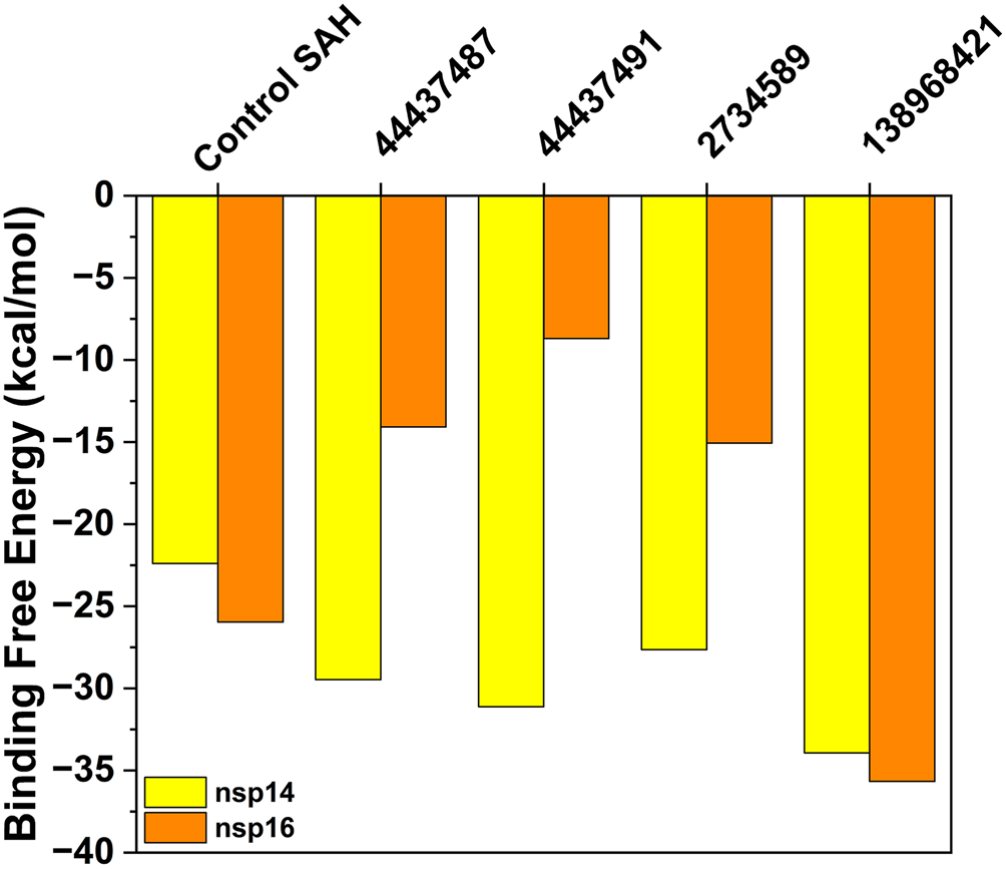
Plot depicting calculated binding free energies of the hit compounds and SAH/SFG with nsp14 and nsp16 protein by MM-GBSA calculation. (SAH = co-crystal of nsp14 and SFG = co-crystal of nsp16).

In order to determine the binding free energy between the bound state (complex form) and entirely unbound states (compound removed from the binding pocket of the protein) for all compounds, MM-GBSA calculations were conducted. In the case of nsp14 protein, all four compounds exhibited more negative total Gibbs free energy (ΔG_Total_) values (ranging from − 27.65 to − 33.94 kcal/mol) compared to SAH − 22.4 kcal/mol) (Fig. 7). This suggests that these hit compounds possess a greater affinity for binding towards nsp14 protein in comparison to SAH. Amongst all, compound 138968421 exhibited the higher negative energy (− 33.94 kcal/mol) suggesting its most efficient binding with the nsp14 protein. In the case of nsp16, ΔG_Total_ was calculated to be in the range of − 8.7 kcal/mol to − 35.67 kcal/mol, whereas SFG showed ΔG_Total_ = − 25.96 kcal/mol. The compound 138968421 showed the highest negative energy with a score of − 35.67 kcal/mol indicating its efficient binding with the nsp16 protein.

**Figure 8:**
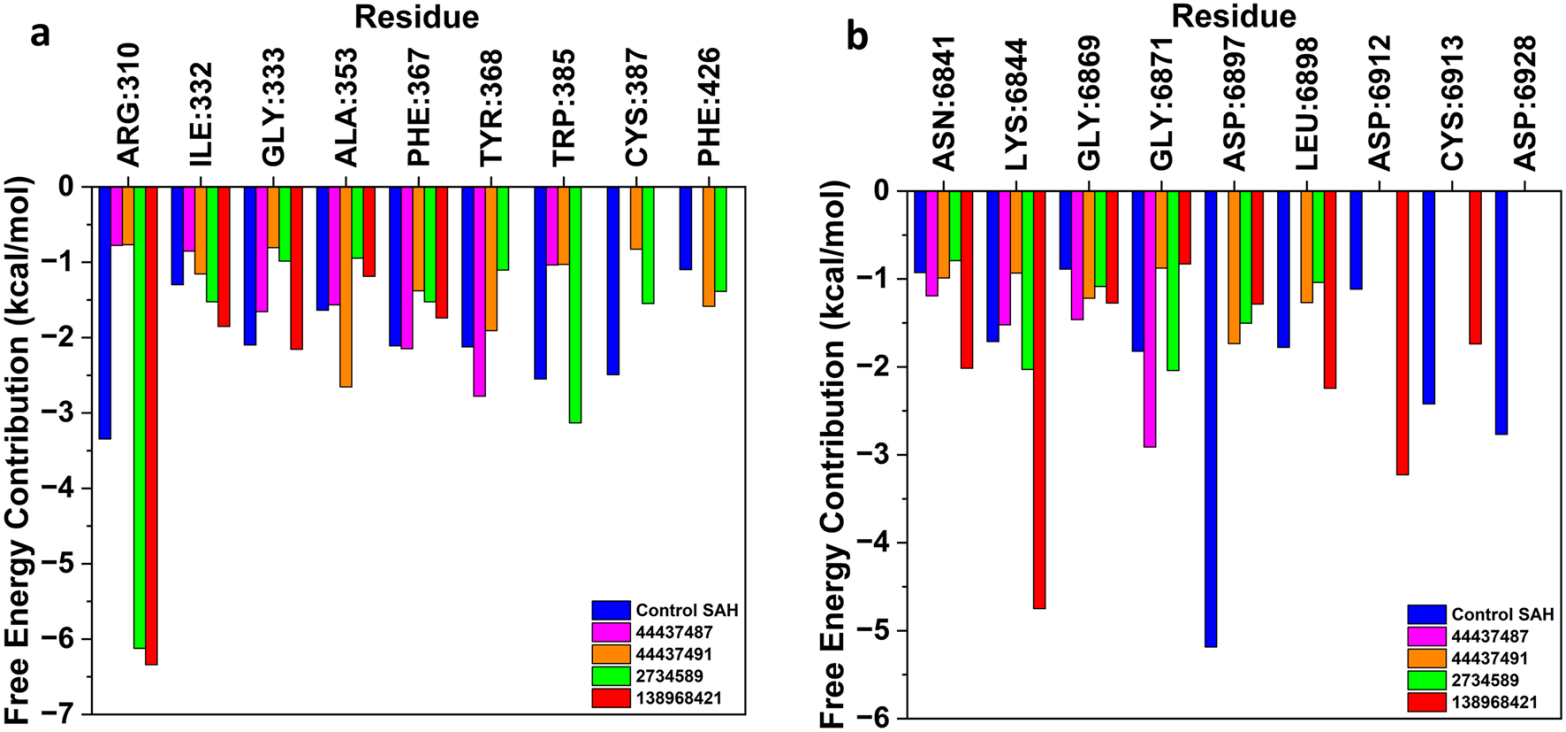
Plot depicting the hot residues in the binding pocket of (**a**) nsp14 and (**b**) nsp16 protein upon interaction with control SAH/SFG, 44437487, 44437491, 2734589 and 138968421.

Further, to identify the crucial residues that contribute to the binding affinity at the nsp14 and nsp16 protein's binding pocket, decomposition analysis was carried out. It is well known that an amino acid residue is considered important for inhibiting activity against the target protein if the energy of interaction between the ligand and that residue is lower than − 1 kcal/mol and this residue is referred to as a “hot residue”^41,42^. The decomposition energy contributions of some hot residues in case of nsp14 (Arg310, Ile332, Gly333, Ala353, and Phe367) and nsp16 (Asn6841, Lys6844, Gly6869, Gly6871) with all the compounds are depicted in Fig. 8. The decomposition analysis of nsp14 protein was found to be consistent with the molecular docking and time-dependent MD simulation results and indicates that all the compounds stayed at the binding pocket of the protein during the simulation period. On the other hand, in case of nsp16 protein, certain deviations were observed from the binding pocket of the protein which corroborates with the time-dependent MD simulations.

### Electronic structure and molecular orbital calculations for hit compounds

**Figure 9:**
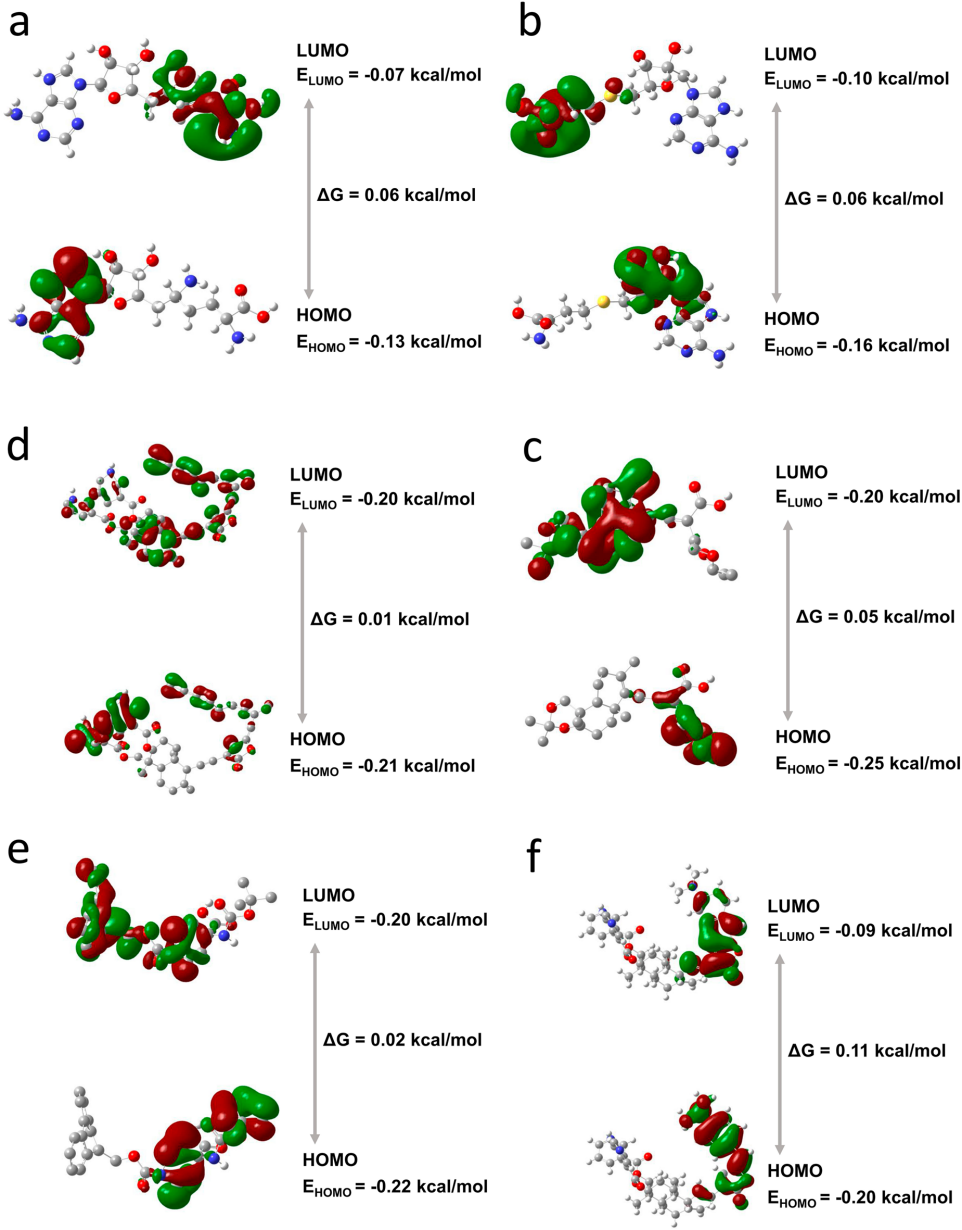
3D depiction of frontier molecular orbitals (FMOs) with band gap energy for the compounds (**a**) SAH (**b**) SFG (**c**) 44437487, (**d**) 44437491, (**e**) 2734589 and (**f**) 138968421.

For a deeper understanding of the chemical reactivity of the hit compounds towards the binding pocket of the protein, an analysis of frontier molecular orbitals (FMO) was conducted. The compounds 44437491 and 44437487 exhibited the least negative total energy, measuring − 2203.5571 kcal/mol and − 2159.26 kcal/mol respectively, suggesting their stronger binding affinity with the binding pocket of the protein. The remaining two compounds showed a total energy value of − 1440.19 kcal/mol (138968421) and − 1550.90 kcal/mol (2734589) which was higher than the control SAH (− 1647.96 kcal/mol) and lower than control SFG (− 1344.65 kcal/mol). The presence of more negative values indicates a favourable binding affinity between the hit compounds and the protein moiety. Further, the hit compounds stability in relation to the protein was assessed by computing the band gap energy (ΔG), which is derived from the difference between the energy levels of the lowest unoccupied molecular orbital (E_LUMO_) and the highest occupied molecular orbital (E_HOMO_). The molecule 44437487 exhibited the smallest band gap value of 0.01 kcal/mol, whereas larger values were found for 44437491 (0.11 kcal/mol), 2734589 (0.02 kcal/mol), 138968421 (0.06 kcal/mol), SAH (0.06 kcal/mol), and SFG (0.06 kcal/mol) (Fig. 9). This suggests that molecule 44437487 exhibits greater chemical reactivity in comparison to the other hit compounds. Additionally, the ability of hydrogen bond formation was evaluated by computing dipole moment. The results indicated that compound 44437487 has the highest value of 16.06, whereas compounds 44437491 (8.77), 2734589 (9.09), 138968421 (3.25), SAH (11.26), and SFG (7.33) display lower values. These findings are in good agreement with the results drawn from the molecular docking and MD simulation analyses and further establish the prominent role of hydrophobic and electrostatic interactions in the binding of the hit compounds to the protein.

**Figure 10:**
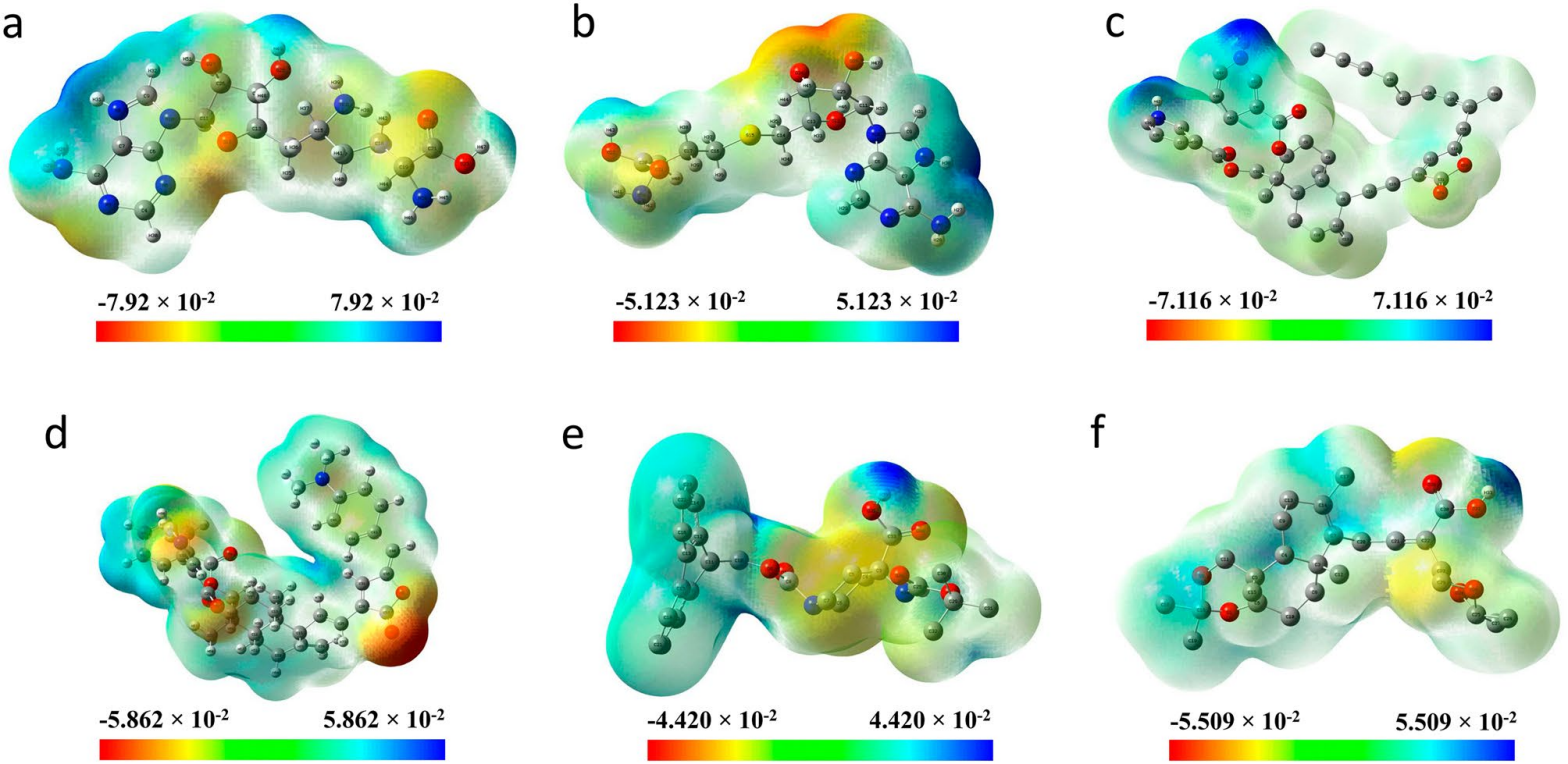
Molecular electronic potential (MEP) maps for the compounds (**a**) SAH (**b**) SFG (**c**) 44437487 (**d**) 44437491 (**e**) 2734589 and (**f**) 138968421.

The electrostatic potential for the hit compounds was determined by examining the molecular electrostatic potential (MEP) maps which provide valuable information regarding the spatial distribution of charges within compounds and thereby facilitate the analysis of their interactions with amino acids within proteins. The regions with high electronegativity, representing the negative extreme, are illustrated in red, while the regions with high electropositivity, representing the positive extreme, are depicted in blue. The neutral regions, which have the ability to engage in hydrophobic interactions, are displayed in shades ranging from yellow to green. Figure 10 illustrates the presence of visually comparable red and blue zones for all four hit compounds and SAH and SFG. This observation indicates the possibility of the hit compounds to form hydrogen bonds. Moreover, the SAH exhibited higher negative (− 0.079 a.u.) and positive (0.079 a.u.) extremes, indicating a greater possibility of hydrogen bond formation. This observation aligns well with the findings of the molecular docking investigation. The compound 44437487 demonstrated comparable extremes as of SAH (− 0.071 a.u. to 0.071 a.u.) which indicates its hydrogen bond forming capability. In contrast, the other hit compounds exhibited more neutral regions compared to electronegative/electropositive regions, suggesting that their interaction is mostly driven by hydrophobic contacts rather than hydrogen bonding.

Amongst all the andrographolide derivatives investigated, compounds 2734589 and 138968421 showed the potential as effective natural drug candidate against both nsp14 and nsp16 proteins due to their strong interaction primarily via hydrophobic and hydrogen bonding. A much lower binding energy and thereby greater affinity was observed for both the compounds compared to other reported natural compounds like amentoflavone (− 9.3 kcal/mol), baicalin (− 8.7 kcal/mol), daidzin (− 8.3 kcal/mol), luteoloside (− 8.3 kcal/mol), machaeriols (− 8.9 kcal/mol)^17,18^. Further, these reported lead compounds are majorly stabilized by hydrogen bonds which are in good resonance with both the compounds identified in our study. However, in our study, both the compounds showed a significant number of hydrophobic and electrostatic interactions which results in more negative binding energy value and effective interaction with the proteins. Further, on comparing the molecular dynamic trajectory, both compounds, 2734589 and 138968421, showed comparable results with that of the earlier reported natural compounds. Although, our results showed slightly higher average trajectory values (i.e. RMSD, RMSF, Rg, SASA and hydrogen bond) compared to the lead natural compounds reported in an earlier study, both the compounds remained intact at the binding pocket of the MTs as confirmed from the time-dependent MD study^18^. Overall, in-depth cheminformatics analysis in the present study establishes the compounds 2734589 and 138968421 as potential drug-like molecules that can inhibit the methyltransferases of SARS-CoV-2 and help in the therapeutic management of Covid-19 infection. Although computational study predicts the potential drug-like molecules based on well-established algorithms and parameters, the biological activity may differ to some extent. Therefore, the potential clinical implications of the compounds 2734589 and 138968421, need to be assessed as a future perspective via in-vitro and in-vivo analyses to establish the bioactivity and safety of the molecules. In parallel, drug formulation studies need to be carried out to develop an effective dosage form for drugs to be effectively delivered.

## Conclusion

In conclusion, this research has identified a promising new andrographolide derivative that has significant activity against the nsp14 and nsp16 proteins, which may be crucial for preventing the replication of SARS-CoV-2 by suppressing methyltransferase activity. A comprehensive examination was conducted utilizing different cheminformatics approaches, including virtual screening, molecular docking, ADMET prediction, MD simulation, and DFT research, in order to forecast the binding affinity and stability of potential compounds with nsp14 and nsp16 proteins. Interestingly, compounds 2734589 and 138968421 have shown to have good affinity towards both the protein targets with better stability and non-toxic nature. This observation strongly indicates that the activity of both the andrographolide derivatives would inhibit the functioning of nsp14 and nsp16, ultimately resulting in a reduction of viral load within the host. In general, this study has yielded valuable insights into the atomic-level inhibition of nsp14 and nsp16 proteins, as well as the identification of a novel natural bioactive andrographolide derivative. Further, it is anticipated that the outcomes of this study will provide valuable insights to the scientific community in the development of innovative pharmaceutical interventions for combating SARS-CoV-2 infection.

### Supplementary Information

Supplementary Information.

## Data Availability

The datasets used and/or analyzed during the current study available from the corresponding author on reasonable request.
