# Supplementary material for: Cheminformatics approach to identify andrographolide derivatives as dual inhibitors of methyltransferases (nsp14 and nsp16) of SARS-CoV-2
Source: Sci Rep. 2024 Apr 29;14:9801. doi: 10.1038/s41598-024-58532-7 (PMC11058777; doi:10.1038/s41598-024-58532-7)
Supplement: Supplementary file 1 — Supplementary Information. [file 41598_2024_58532_MOESM1_ESM.docx]

**Supplementary Information**

**Cheminformatics Approach to Identify Andrographolide Derivatives as Dual Inhibitors of Methyltransferases (nsp14 and nsp16) of SARS-CoV-2**

Jobin Thomas^1^_,_ Anupam Ghosh^2^, Shivendu Ranjan^2^, Jitendra Satija^1*^

^1^ Centre for Nanobiotechnology (CNBT), Vellore Institute of Technology, Vellore, Tamil Nadu, India 632014

^2^ NanoBio Research Lab, School of Nano Science and Technology, Indian Institute of Technology Kharagpur, Kharagpur, West Bengal, India 721301

* Corresponding author: [jsatija11@gmail.com](mailto:jsatija11@gmail.com) (Dr. J. Satija)

**Table S1.** Overview of the binding energy of the compounds based on virtual screening with nsp14 protein

| S. No. | Ligand | Binding Affinity |
| --- | --- | --- |
| **1** | **44437491** | **-11.4** |
| **2** | **44437493** | **-11.3** |
| **3** | **44437487** | **-10.9** |
| **4** | **145069497** | **-10.9** |
| **5** | **44437492** | **-10.9** |
| **6** | **46906680** | **-10.3** |
| **7** | **46906636** | **-11.5** |
| **8** | **138481435** | **-10.9** |
| **9** | **2734589** | **-11.1** |
| **10** | **138968421** | **-10.3** |
| 11 | 70691198 | -10.1 |
| 12 | 42646332 | -10.1 |
| 13 | 44437486 | -10.1 |
| 14 | 58723239 | -10.1 |
| 15 | 139070844 | -10 |
| 16 | 72401645 | -10 |
| 17 | 140434890 | -9.9 |
| 18 | 142079580 | -9.9 |
| 19 | 70682784 | -9.9 |
| 20 | 118108006 | -9.8 |
| 21 | 123610739 | -9.8 |
| 22 | 72401643 | -9.8 |
| 23 | 52950159 | -9.7 |
| 24 | 146157060 | -9.6 |
| 25 | 59453160 | -9.6 |
| 26 | 75238669 | -9.6 |
| 27 | 89809110 | -9.6 |
| 28 | 90783400 | -9.6 |
| 29 | 90922728 | -9.6 |
| 30 | 91317586 | -9.6 |
| 31 | 132223213 | -9.5 |
| 32 | 138968359 | -9.5 |
| 33 | 50908714 | -9.5 |
| 34 | 52947253 | -9.5 |
| 35 | 10567584 | -9.4 |
| 36 | 129012165 | -9.4 |
| 37 | 132210481 | -9.4 |
| 38 | 154495958 | -9.4 |
| 39 | 16122493 | -9.4 |
| 40 | 162877452 | -9.4 |
| 41 | 162877454 | -9.4 |
| 42 | 46871816 | -9.4 |
| 43 | 46906151 | -9.4 |
| 44 | 71550939 | -9.4 |
| 45 | 91034308 | -9.4 |
| 46 | 101362374 | -9.3 |
| 47 | 101476459 | -9.3 |
| 48 | 129012173 | -9.3 |
| 49 | 132217478 | -9.3 |
| 50 | 132217497 | -9.3 |
| 51 | 132217570 | -9.3 |
| 52 | 132217578 | -9.3 |
| 53 | 132223154 | -9.3 |
| 54 | 16122395 | -9.3 |
| 55 | 44411130 | -9.3 |
| 56 | 72506758 | -9.3 |
| 57 | 90710032 | -9.3 |
| 58 | 10041596 | -9.2 |
| 59 | 102271960 | -9.2 |
| 60 | 102463322 | -9.2 |
| 61 | 11099202 | -9.2 |
| 62 | 123361152 | -9.2 |
| 63 | 127040078 | -9.2 |
| 64 | 132210355 | -9.2 |
| 65 | 132210372 | -9.2 |
| 66 | 132210373 | -9.2 |
| 67 | 132210552 | -9.2 |
| 68 | 132210553 | -9.2 |
| 69 | 132217499 | -9.2 |
| 70 | 132223170 | -9.2 |
| 71 | 143270556 | -9.2 |
| 72 | 145069422 | -9.2 |
| 73 | 145069454 | -9.2 |
| 74 | 145956786 | -9.2 |
| 75 | 147144082 | -9.2 |
| 76 | 16664361 | -9.2 |
| 77 | 25154345 | -9.2 |
| 78 | 44409284 | -9.2 |
| 79 | 58209855 | -9.2 |
| 80 | 59741079 | -9.2 |
| 81 | 59876490 | -9.2 |
| 82 | 59921242 | -9.2 |
| 83 | 68402469 | -9.2 |
| 84 | 73357050 | -9.2 |
| 85 | 90796586 | -9.2 |
| 86 | 118108005 | -9.1 |
| 87 | 118712682 | -9.1 |
| 88 | 132217514 | -9.1 |
| 89 | 132217515 | -9.1 |
| 90 | 132217538 | -9.1 |
| 91 | 142095947 | -9.1 |
| 92 | 160301931 | -9.1 |
| 93 | 21679044 | -9.1 |
| 94 | 44575272 | -9.1 |
| 95 | 59921241 | -9.1 |
| 96 | 6373011 | -9.1 |
| 97 | 71578897 | -9.1 |
| 98 | 72191643 | -9.1 |
| 99 | 11869596 | -9 |
| 100 | 124604719 | -9 |
| 101 | 132210370 | -9 |
| 102 | 132210408 | -9 |
| 103 | 132210409 | -9 |
| 104 | 132210460 | -9 |
| 105 | 132210461 | -9 |
| 106 | 132210478 | -9 |
| 107 | 132210512 | -9 |
| 108 | 132210551 | -9 |
| 109 | 132217516 | -9 |
| 110 | 132217517 | -9 |
| 111 | 132217523 | -9 |
| 112 | 135339861 | -9 |
| 113 | 162869835 | -9 |
| 114 | 163010980 | -9 |
| 115 | 163010983 | -9 |
| 116 | 163273906 | -9 |
| 117 | 25566761 | -9 |
| 118 | 44411016 | -9 |
| 119 | 57395013 | -9 |
| 120 | 59881738 | -9 |
| 121 | 59897190 | -9 |
| 122 | 6875366 | -9 |
| 123 | 6978717 | -9 |
| 124 | 7061135 | -9 |
| 125 | 71589914 | -9 |
| 126 | 73351017 | -9 |
| 127 | 90765060 | -9 |
| 128 | 91066252 | -9 |
| 129 | 91499723 | -9 |
| 130 | 92215519 | -9 |
| 131 | 92218179 | -9 |
| 132 | 92258406 | -9 |
| 133 | 11717280 | -8.9 |
| 134 | 11876149 | -8.9 |
| 135 | 1268030 | -8.9 |
| 136 | 127042688 | -8.9 |
| 137 | 127042992 | -8.9 |
| 138 | 129010070 | -8.9 |
| 139 | 129360331 | -8.9 |
| 140 | 129360344 | -8.9 |
| 141 | 132210396 | -8.9 |
| 142 | 132210482 | -8.9 |
| 143 | 132210507 | -8.9 |
| 144 | 132210549 | -8.9 |
| 145 | 132217489 | -8.9 |
| 146 | 132223104 | -8.9 |
| 147 | 162949476 | -8.9 |
| 148 | 162949477 | -8.9 |
| 149 | 163010982 | -8.9 |
| 150 | 163046217 | -8.9 |
| 151 | 163193646 | -8.9 |
| 152 | 16401724 | -8.9 |
| 153 | 25114900 | -8.9 |
| 154 | 57391527 | -8.9 |
| 155 | 57403747 | -8.9 |
| 156 | 7061133 | -8.9 |
| 157 | 91938222 | -8.9 |
| 158 | 95359254 | -8.9 |
| 159 | 95359255 | -8.9 |
| 160 | 11870301 | -8.8 |
| 161 | 11870302 | -8.8 |
| 162 | 11870303 | -8.8 |
| 163 | 124338905 | -8.8 |
| 164 | 124677506 | -8.8 |
| 165 | 127040428 | -8.8 |
| 166 | 129012155 | -8.8 |
| 167 | 130324504 | -8.8 |
| 168 | 131706270 | -8.8 |
| 169 | 132210368 | -8.8 |
| 170 | 132210388 | -8.8 |
| 171 | 132210390 | -8.8 |
| 172 | 132210477 | -8.8 |
| 173 | 132210515 | -8.8 |
| 174 | 132210516 | -8.8 |
| 175 | 132223164 | -8.8 |
| 176 | 145069445 | -8.8 |
| 177 | 145069526 | -8.8 |
| 178 | 145316789 | -8.8 |
| 179 | 145953545 | -8.8 |
| 180 | 154790511 | -8.8 |
| 181 | 15922987 | -8.8 |
| 182 | 163010981 | -8.8 |
| 183 | 163010984 | -8.8 |
| 184 | 163010985 | -8.8 |
| 185 | 16406675 | -8.8 |
| 186 | 16758033 | -8.8 |
| 187 | 1777573 | -8.8 |
| 188 | 24957530 | -8.8 |
| 189 | 28362188 | -8.8 |
| 190 | 28362190 | -8.8 |
| 191 | 40561086 | -8.8 |
| 192 | 42556578 | -8.8 |
| 193 | 42556579 | -8.8 |
| 194 | 44409292 | -8.8 |
| 195 | 44409328 | -8.8 |
| 196 | 46907192 | -8.8 |
| 197 | 46917415 | -8.8 |
| 198 | 51055161 | -8.8 |
| 199 | 53298396 | -8.8 |
| 200 | 5458931 | -8.8 |
| 201 | 59741075 | -8.8 |
| 202 | 59876519 | -8.8 |
| 203 | 59897192 | -8.8 |
| 204 | 59897200 | -8.8 |
| 205 | 6436016 | -8.8 |
| 206 | 6572456 | -8.8 |
| 207 | 6713120 | -8.8 |
| 208 | 6857767 | -8.8 |
| 209 | 7067322 | -8.8 |
| 210 | 7067323 | -8.8 |
| 211 | 71308151 | -8.8 |
| 212 | 71589898 | -8.8 |
| 213 | 72166961 | -8.8 |
| 214 | 90718614 | -8.8 |
| 215 | 98803586 | -8.8 |
| 216 | 98803587 | -8.8 |
| 217 | 99719588 | -8.8 |
| 218 | 99719589 | -8.8 |
| 219 | 118712679 | -8.7 |
| 220 | 123430276 | -8.7 |
| 221 | 129012148 | -8.7 |
| 222 | 129012174 | -8.7 |
| 223 | 132210364 | -8.7 |
| 224 | 132210395 | -8.7 |
| 225 | 132217527 | -8.7 |
| 226 | 132217529 | -8.7 |
| 227 | 145970059 | -8.7 |
| 228 | 161951754 | -8.7 |
| 229 | 44411049 | -8.7 |
| 230 | 44411126 | -8.7 |
| 231 | 44575278 | -8.7 |
| 232 | 46907273 | -8.7 |
| 233 | 46907311 | -8.7 |
| 234 | 46907313 | -8.7 |
| 235 | 57401998 | -8.7 |
| 236 | 59876515 | -8.7 |
| 237 | 59876522 | -8.7 |
| 238 | 6473762 | -8.7 |
| 239 | 10065491 | -8.6 |
| 240 | 11078630 | -8.6 |
| 241 | 11624798 | -8.6 |
| 242 | 117673341 | -8.6 |
| 243 | 118122636 | -8.6 |
| 244 | 11869597 | -8.6 |
| 245 | 123179837 | -8.6 |
| 246 | 123377869 | -8.6 |
| 247 | 123516411 | -8.6 |
| 248 | 124040756 | -8.6 |
| 249 | 127040079 | -8.6 |
| 250 | 129010368 | -8.6 |
| 251 | 129012163 | -8.6 |
| 252 | 129316784 | -8.6 |
| 253 | 132223040 | -8.6 |
| 254 | 145953671 | -8.6 |
| 255 | 145956444 | -8.6 |
| 256 | 157411829 | -8.6 |
| 257 | 15922990 | -8.6 |
| 258 | 162922963 | -8.6 |
| 259 | 163186069 | -8.6 |
| 260 | 163187150 | -8.6 |
| 261 | 1777572 | -8.6 |
| 262 | 23239822 | -8.6 |
| 263 | 24206412 | -8.6 |
| 264 | 24980082 | -8.6 |
| 265 | 25114469 | -8.6 |
| 266 | 343585 | -8.6 |
| 267 | 5351335 | -8.6 |
| 268 | 59741104 | -8.6 |
| 269 | 59881734 | -8.6 |
| 270 | 65153 | -8.6 |
| 271 | 6708647 | -8.6 |
| 272 | 7061132 | -8.6 |
| 273 | 7061134 | -8.6 |
| 274 | 7067324 | -8.6 |
| 275 | 7067325 | -8.6 |
| 276 | 7067837 | -8.6 |
| 277 | 71307452 | -8.6 |
| 278 | 71590622 | -8.6 |
| 279 | 72191955 | -8.6 |
| 280 | 73265376 | -8.6 |
| 281 | 77410137 | -8.6 |
| 282 | 90985283 | -8.6 |
| 283 | 91032405 | -8.6 |
| 284 | 91221436 | -8.6 |
| 285 | 91268827 | -8.6 |
| 286 | 91358749 | -8.6 |
| 287 | 91408358 | -8.6 |
| 288 | 9998023 | -8.6 |
| 289 | 102173547 | -8.5 |
| 290 | 11624161 | -8.5 |
| 291 | 118566340 | -8.5 |
| 292 | 118712676 | -8.5 |
| 293 | 123197430 | -8.5 |
| 294 | 132210479 | -8.5 |
| 295 | 132217511 | -8.5 |
| 296 | 132223153 | -8.5 |
| 297 | 142095931 | -8.5 |
| 298 | 145069449 | -8.5 |
| 299 | 145957487 | -8.5 |
| 300 | 145965103 | -8.5 |
| 301 | 154578334 | -8.5 |
| 302 | 20054828 | -8.5 |
| 303 | 23757051 | -8.5 |
| 304 | 44393882 | -8.5 |
| 305 | 44575273 | -8.5 |
| 306 | 44575279 | -8.5 |
| 307 | 452937 | -8.5 |
| 308 | 5318517 | -8.5 |
| 309 | 5708351 | -8.5 |
| 310 | 59881735 | -8.5 |
| 311 | 59897195 | -8.5 |
| 312 | 59897206 | -8.5 |
| 313 | 102007111 | -8.4 |
| 314 | 129317078 | -8.4 |
| 315 | 132210365 | -8.4 |
| 316 | 132217526 | -8.4 |
| 317 | 138059743 | -8.4 |
| 318 | 145069455 | -8.4 |
| 319 | 145069510 | -8.4 |
| 320 | 146158138 | -8.4 |
| 321 | 162669031 | -8.4 |
| 322 | 162908957 | -8.4 |
| 323 | 16394566 | -8.4 |
| 324 | 25121277 | -8.4 |
| 325 | 363866 | -8.4 |
| 326 | 44393895 | -8.4 |
| 327 | 44574537 | -8.4 |
| 328 | 57395012 | -8.4 |
| 329 | 59070294 | -8.4 |
| 330 | 59897197 | -8.4 |
| 331 | 59897198 | -8.4 |
| 332 | 68103125 | -8.4 |
| 333 | 68103289 | -8.4 |
| 334 | 71624124 | -8.4 |
| 335 | 73661245 | -8.4 |
| 336 | 75022103 | -8.4 |
| 337 | 10914618 | -8.3 |
| 338 | 118712674 | -8.3 |
| 339 | 132217520 | -8.3 |
| 340 | 137432754 | -8.3 |
| 341 | 145069500 | -8.3 |
| 342 | 153176606 | -8.3 |
| 343 | 21679042 | -8.3 |
| 344 | 23757050 | -8.3 |
| 345 | 24879275 | -8.3 |
| 346 | 24879276 | -8.3 |
| 347 | 38350563 | -8.3 |
| 348 | 38350572 | -8.3 |
| 349 | 44394021 | -8.3 |
| 350 | 44575263 | -8.3 |
| 351 | 59143923 | -8.3 |
| 352 | 68169653 | -8.3 |
| 353 | 102463323 | -8.2 |
| 354 | 10450540 | -8.2 |
| 355 | 132210480 | -8.2 |
| 356 | 132217521 | -8.2 |
| 357 | 145966285 | -8.2 |
| 358 | 154790154 | -8.2 |
| 359 | 16038718 | -8.2 |
| 360 | 29927575 | -8.2 |
| 361 | 44393988 | -8.2 |
| 362 | 44437436 | -8.2 |
| 363 | 86578936 | -8.2 |
| 364 | 90870076 | -8.2 |
| 365 | 11382524 | -8.1 |
| 366 | 132210508 | -8.1 |
| 367 | 44393868 | -8.1 |
| 368 | 44393935 | -8.1 |
| 369 | 46907271 | -8.1 |
| 370 | 58723234 | -8.1 |
| 371 | 59070369 | -8.1 |
| 372 | 71589913 | -8.1 |
| 373 | 12116585 | -8 |
| 374 | 139258927 | -8 |
| 375 | 142095944 | -8 |
| 376 | 145069431 | -8 |
| 377 | 145069444 | -8 |
| 378 | 145069458 | -8 |
| 379 | 162940935 | -8 |
| 380 | 44393828 | -8 |
| 381 | 59070301 | -8 |
| 382 | 59872000 | -8 |
| 383 | 59876516 | -8 |
| 384 | 60147888 | -8 |
| 385 | 71624251 | -8 |
| 386 | 73353957 | -8 |
| 387 | 145957890 | -7.9 |
| 388 | 146158882 | -7.9 |
| 389 | 44393859 | -7.9 |
| 390 | 90676608 | -7.9 |
| 391 | 142095942 | -7.8 |
| 392 | 59876523 | -7.8 |
| 393 | 132212629 | -7.7 |
| 394 | 138968420 | -7.7 |
| 395 | 118712675 | -7.6 |
| 396 | 145720760 | -7.5 |
| 397 | 44575277 | -7.5 |
| 398 | 59876491 | -7.5 |
| 399 | 56776171 | -7.3 |
| 400 | 57403993 | -7.3 |
| 401 | 44393932 | -7.2 |
| 402 | 139258929 | -6.5 |
| 403 | 24178376 | -5.8 |
| 404 | 139258930 | -5.4 |
| 405 | 139258931 | -5.2 |
| 406 | 53436107 | -5.2 |
| 407 | 143607623 | -5.1 |
| 408 | 68796320 | -4.9 |
| 409 | 72194390 | -3.3 |
| 410 | 157746974 | -1.1 |
| 411 | 157887226 | -1.1 |
| 412 | 160619786 | -1.1 |
| 413 | 162237387 | -1.1 |

**Table S2.** Overview of the binding energy of the compounds based on virtual screening with nsp16 protein

| S. No. | Ligand | Binding Affinity |
| --- | --- | --- |
| **1** | **138968421** | **-8.4** |
| **2** | **2734589** | **-8.4** |
| **3** | **44437491** | **-8.4** |
| **4** | **127042688** | **-8.3** |
| **5** | **44437487** | **-8.3** |
| **6** | **10450540** | **-8.2** |
| **7** | **11382524** | **-8.2** |
| **8** | **132210355** | **-8.2** |
| **9** | **132210552** | **-8.2** |
| **10** | **132210553** | **-8.2** |
| 11 | 132223170 | -8.2 |
| 12 | 59070369 | -8.2 |
| 13 | 132210516 | -8.1 |
| 14 | 132210549 | -8.1 |
| 15 | 44411126 | -8 |
| 16 | 58723234 | -8 |
| 17 | 58723239 | -8 |
| 18 | 72401643 | -8 |
| 19 | 72401645 | -8 |
| 20 | 127040079 | -7.9 |
| 21 | 132210515 | -7.9 |
| 22 | 138481435 | -7.9 |
| 23 | 145069497 | -7.9 |
| 24 | 46906636 | -7.9 |
| 25 | 59897206 | -7.9 |
| 26 | 71589898 | -7.9 |
| 27 | 11624798 | -7.8 |
| 28 | 142095942 | -7.8 |
| 29 | 145069449 | -7.8 |
| 30 | 154790154 | -7.8 |
| 31 | 163186069 | -7.8 |
| 32 | 44437493 | -7.8 |
| 33 | 46906680 | -7.8 |
| 34 | 59876523 | -7.8 |
| 35 | 73357050 | -7.8 |
| 36 | 132210388 | -7.7 |
| 37 | 132210396 | -7.7 |
| 38 | 132210479 | -7.7 |
| 39 | 132223213 | -7.7 |
| 40 | 138968359 | -7.7 |
| 41 | 139070844 | -7.7 |
| 42 | 16664361 | -7.7 |
| 43 | 24206412 | -7.7 |
| 44 | 44437492 | -7.7 |
| 45 | 58209855 | -7.7 |
| 46 | 59921242 | -7.7 |
| 47 | 68402469 | -7.7 |
| 48 | 72506758 | -7.7 |
| 49 | 90710032 | -7.7 |
| 50 | 90796586 | -7.7 |
| 51 | 9998023 | -7.7 |
| 52 | 101362374 | -7.6 |
| 53 | 118108005 | -7.6 |
| 54 | 123361152 | -7.6 |
| 55 | 123430276 | -7.6 |
| 56 | 132210390 | -7.6 |
| 57 | 132210409 | -7.6 |
| 58 | 44575279 | -7.6 |
| 59 | 70691198 | -7.6 |
| 60 | 118108006 | -7.5 |
| 61 | 12116585 | -7.5 |
| 62 | 129012173 | -7.5 |
| 63 | 132223164 | -7.5 |
| 64 | 145069445 | -7.5 |
| 65 | 16122395 | -7.5 |
| 66 | 16122493 | -7.5 |
| 67 | 44411049 | -7.5 |
| 68 | 59881734 | -7.5 |
| 69 | 68103289 | -7.5 |
| 70 | 90870076 | -7.5 |
| 71 | 10065491 | -7.4 |
| 72 | 123610739 | -7.4 |
| 73 | 127040428 | -7.4 |
| 74 | 127042992 | -7.4 |
| 75 | 129012148 | -7.4 |
| 76 | 129012174 | -7.4 |
| 77 | 132210477 | -7.4 |
| 78 | 132217489 | -7.4 |
| 79 | 135339861 | -7.4 |
| 80 | 142095947 | -7.4 |
| 81 | 145953671 | -7.4 |
| 82 | 153176606 | -7.4 |
| 83 | 25114900 | -7.4 |
| 84 | 44411130 | -7.4 |
| 85 | 57395012 | -7.4 |
| 86 | 57395013 | -7.4 |
| 87 | 59143923 | -7.4 |
| 88 | 59897190 | -7.4 |
| 89 | 7061132 | -7.4 |
| 90 | 71307452 | -7.4 |
| 91 | 72191955 | -7.4 |
| 92 | 90783400 | -7.4 |
| 93 | 91066252 | -7.4 |
| 94 | 91408358 | -7.4 |
| 95 | 102173547 | -7.3 |
| 96 | 117673341 | -7.3 |
| 97 | 118566340 | -7.3 |
| 98 | 11869597 | -7.3 |
| 99 | 130324504 | -7.3 |
| 100 | 132210408 | -7.3 |
| 101 | 132217514 | -7.3 |
| 102 | 138059743 | -7.3 |
| 103 | 145069422 | -7.3 |
| 104 | 145069444 | -7.3 |
| 105 | 145069454 | -7.3 |
| 106 | 145069455 | -7.3 |
| 107 | 157411829 | -7.3 |
| 108 | 15922990 | -7.3 |
| 109 | 1777572 | -7.3 |
| 110 | 20054828 | -7.3 |
| 111 | 25121277 | -7.3 |
| 112 | 44575273 | -7.3 |
| 113 | 44575278 | -7.3 |
| 114 | 52947253 | -7.3 |
| 115 | 59876515 | -7.3 |
| 116 | 68103125 | -7.3 |
| 117 | 7061134 | -7.3 |
| 118 | 7067837 | -7.3 |
| 119 | 71590622 | -7.3 |
| 120 | 72191643 | -7.3 |
| 121 | 90676608 | -7.3 |
| 122 | 90985283 | -7.3 |
| 123 | 11099202 | -7.2 |
| 124 | 11717280 | -7.2 |
| 125 | 129012165 | -7.2 |
| 126 | 132210372 | -7.2 |
| 127 | 132210373 | -7.2 |
| 128 | 132217499 | -7.2 |
| 129 | 132217515 | -7.2 |
| 130 | 132217538 | -7.2 |
| 131 | 132223153 | -7.2 |
| 132 | 145956786 | -7.2 |
| 133 | 161951754 | -7.2 |
| 134 | 162949476 | -7.2 |
| 135 | 162949477 | -7.2 |
| 136 | 25114469 | -7.2 |
| 137 | 44393859 | -7.2 |
| 138 | 452937 | -7.2 |
| 139 | 46907313 | -7.2 |
| 140 | 59741104 | -7.2 |
| 141 | 71624251 | -7.2 |
| 142 | 91221436 | -7.2 |
| 143 | 91268827 | -7.2 |
| 144 | 102271960 | -7.1 |
| 145 | 11869596 | -7.1 |
| 146 | 11870301 | -7.1 |
| 147 | 11870302 | -7.1 |
| 148 | 11870303 | -7.1 |
| 149 | 11876149 | -7.1 |
| 150 | 124338905 | -7.1 |
| 151 | 124604719 | -7.1 |
| 152 | 124677506 | -7.1 |
| 153 | 1268030 | -7.1 |
| 154 | 129010070 | -7.1 |
| 155 | 129360331 | -7.1 |
| 156 | 129360344 | -7.1 |
| 157 | 131706270 | -7.1 |
| 158 | 132210508 | -7.1 |
| 159 | 132217478 | -7.1 |
| 160 | 132217511 | -7.1 |
| 161 | 132217526 | -7.1 |
| 162 | 145069431 | -7.1 |
| 163 | 145069500 | -7.1 |
| 164 | 146157060 | -7.1 |
| 165 | 146158138 | -7.1 |
| 166 | 154790511 | -7.1 |
| 167 | 15922987 | -7.1 |
| 168 | 162922963 | -7.1 |
| 169 | 162940935 | -7.1 |
| 170 | 163010980 | -7.1 |
| 171 | 163010981 | -7.1 |
| 172 | 163010982 | -7.1 |
| 173 | 163010983 | -7.1 |
| 174 | 163010984 | -7.1 |
| 175 | 163046217 | -7.1 |
| 176 | 163273906 | -7.1 |
| 177 | 16401724 | -7.1 |
| 178 | 1777573 | -7.1 |
| 179 | 25566761 | -7.1 |
| 180 | 28362188 | -7.1 |
| 181 | 28362190 | -7.1 |
| 182 | 40561086 | -7.1 |
| 183 | 42556578 | -7.1 |
| 184 | 42556579 | -7.1 |
| 185 | 44411016 | -7.1 |
| 186 | 44437486 | -7.1 |
| 187 | 46917415 | -7.1 |
| 188 | 51055161 | -7.1 |
| 189 | 52950159 | -7.1 |
| 190 | 53298396 | -7.1 |
| 191 | 57403747 | -7.1 |
| 192 | 59881738 | -7.1 |
| 193 | 6572456 | -7.1 |
| 194 | 6713120 | -7.1 |
| 195 | 6875366 | -7.1 |
| 196 | 6978717 | -7.1 |
| 197 | 7061133 | -7.1 |
| 198 | 7061135 | -7.1 |
| 199 | 7067322 | -7.1 |
| 200 | 7067323 | -7.1 |
| 201 | 71308151 | -7.1 |
| 202 | 71550939 | -7.1 |
| 203 | 72166961 | -7.1 |
| 204 | 75238669 | -7.1 |
| 205 | 91938222 | -7.1 |
| 206 | 92215519 | -7.1 |
| 207 | 92218179 | -7.1 |
| 208 | 92258406 | -7.1 |
| 209 | 95359254 | -7.1 |
| 210 | 95359255 | -7.1 |
| 211 | 98803586 | -7.1 |
| 212 | 98803587 | -7.1 |
| 213 | 99719588 | -7.1 |
| 214 | 99719589 | -7.1 |
| 215 | 132210395 | -7 |
| 216 | 132210481 | -7 |
| 217 | 132210512 | -7 |
| 218 | 132210551 | -7 |
| 219 | 132217497 | -7 |
| 220 | 132217516 | -7 |
| 221 | 132217517 | -7 |
| 222 | 132217523 | -7 |
| 223 | 132217529 | -7 |
| 224 | 132223104 | -7 |
| 225 | 140434890 | -7 |
| 226 | 142095931 | -7 |
| 227 | 145316789 | -7 |
| 228 | 145956444 | -7 |
| 229 | 154495958 | -7 |
| 230 | 162869835 | -7 |
| 231 | 163187150 | -7 |
| 232 | 24879275 | -7 |
| 233 | 24879276 | -7 |
| 234 | 24980082 | -7 |
| 235 | 38350563 | -7 |
| 236 | 38350572 | -7 |
| 237 | 44393828 | -7 |
| 238 | 44393895 | -7 |
| 239 | 44409292 | -7 |
| 240 | 44409328 | -7 |
| 241 | 46871816 | -7 |
| 242 | 59070294 | -7 |
| 243 | 65153 | -7 |
| 244 | 10567584 | -6.9 |
| 245 | 10914618 | -6.9 |
| 246 | 11078630 | -6.9 |
| 247 | 11624161 | -6.9 |
| 248 | 118712674 | -6.9 |
| 249 | 123179837 | -6.9 |
| 250 | 123377869 | -6.9 |
| 251 | 123516411 | -6.9 |
| 252 | 127040078 | -6.9 |
| 253 | 129010368 | -6.9 |
| 254 | 129316784 | -6.9 |
| 255 | 132210364 | -6.9 |
| 256 | 132210368 | -6.9 |
| 257 | 132210507 | -6.9 |
| 258 | 132223154 | -6.9 |
| 259 | 142095944 | -6.9 |
| 260 | 145957890 | -6.9 |
| 261 | 147144082 | -6.9 |
| 262 | 162669031 | -6.9 |
| 263 | 162877452 | -6.9 |
| 264 | 162877454 | -6.9 |
| 265 | 162908957 | -6.9 |
| 266 | 163193646 | -6.9 |
| 267 | 16394566 | -6.9 |
| 268 | 16406675 | -6.9 |
| 269 | 16758033 | -6.9 |
| 270 | 21679042 | -6.9 |
| 271 | 23239822 | -6.9 |
| 272 | 23757051 | -6.9 |
| 273 | 343585 | -6.9 |
| 274 | 44575272 | -6.9 |
| 275 | 50908714 | -6.9 |
| 276 | 5458931 | -6.9 |
| 277 | 57391527 | -6.9 |
| 278 | 59741075 | -6.9 |
| 279 | 59897192 | -6.9 |
| 280 | 59897195 | -6.9 |
| 281 | 6436016 | -6.9 |
| 282 | 6473762 | -6.9 |
| 283 | 6857767 | -6.9 |
| 284 | 7067324 | -6.9 |
| 285 | 7067325 | -6.9 |
| 286 | 71589914 | -6.9 |
| 287 | 71624124 | -6.9 |
| 288 | 73265376 | -6.9 |
| 289 | 86578936 | -6.9 |
| 290 | 90718614 | -6.9 |
| 291 | 91032405 | -6.9 |
| 292 | 91317586 | -6.9 |
| 293 | 118122636 | -6.8 |
| 294 | 118712675 | -6.8 |
| 295 | 123197430 | -6.8 |
| 296 | 129012155 | -6.8 |
| 297 | 129012163 | -6.8 |
| 298 | 132210461 | -6.8 |
| 299 | 132212629 | -6.8 |
| 300 | 132217521 | -6.8 |
| 301 | 132217570 | -6.8 |
| 302 | 132217578 | -6.8 |
| 303 | 132223040 | -6.8 |
| 304 | 143270556 | -6.8 |
| 305 | 145069526 | -6.8 |
| 306 | 145953545 | -6.8 |
| 307 | 145965103 | -6.8 |
| 308 | 163010985 | -6.8 |
| 309 | 24957530 | -6.8 |
| 310 | 363866 | -6.8 |
| 311 | 44393988 | -6.8 |
| 312 | 44409284 | -6.8 |
| 313 | 5351335 | -6.8 |
| 314 | 5708351 | -6.8 |
| 315 | 59453160 | -6.8 |
| 316 | 59876516 | -6.8 |
| 317 | 59881735 | -6.8 |
| 318 | 59897200 | -6.8 |
| 319 | 59921241 | -6.8 |
| 320 | 6708647 | -6.8 |
| 321 | 70682784 | -6.8 |
| 322 | 73351017 | -6.8 |
| 323 | 89809110 | -6.8 |
| 324 | 90765060 | -6.8 |
| 325 | 91358749 | -6.8 |
| 326 | 91499723 | -6.8 |
| 327 | 10041596 | -6.7 |
| 328 | 102007111 | -6.7 |
| 329 | 102463322 | -6.7 |
| 330 | 118712676 | -6.7 |
| 331 | 124040756 | -6.7 |
| 332 | 129317078 | -6.7 |
| 333 | 132210460 | -6.7 |
| 334 | 132210480 | -6.7 |
| 335 | 132217520 | -6.7 |
| 336 | 139258927 | -6.7 |
| 337 | 145966285 | -6.7 |
| 338 | 146158882 | -6.7 |
| 339 | 154578334 | -6.7 |
| 340 | 44394021 | -6.7 |
| 341 | 44575263 | -6.7 |
| 342 | 5318517 | -6.7 |
| 343 | 57401998 | -6.7 |
| 344 | 73661245 | -6.7 |
| 345 | 90922728 | -6.7 |
| 346 | 101476459 | -6.6 |
| 347 | 132210482 | -6.6 |
| 348 | 132217527 | -6.6 |
| 349 | 29927575 | -6.6 |
| 350 | 59070301 | -6.6 |
| 351 | 59876490 | -6.6 |
| 352 | 60147888 | -6.6 |
| 353 | 6373011 | -6.6 |
| 354 | 71578897 | -6.6 |
| 355 | 75022103 | -6.6 |
| 356 | 77410137 | -6.6 |
| 357 | 102463323 | -6.5 |
| 358 | 118712679 | -6.5 |
| 359 | 132210365 | -6.5 |
| 360 | 137432754 | -6.5 |
| 361 | 142079580 | -6.5 |
| 362 | 145069510 | -6.5 |
| 363 | 160301931 | -6.5 |
| 364 | 23757050 | -6.5 |
| 365 | 42646332 | -6.5 |
| 366 | 44393935 | -6.5 |
| 367 | 44574537 | -6.5 |
| 368 | 59872000 | -6.5 |
| 369 | 59897197 | -6.5 |
| 370 | 118712682 | -6.4 |
| 371 | 132210370 | -6.4 |
| 372 | 132210478 | -6.4 |
| 373 | 145720760 | -6.4 |
| 374 | 145957487 | -6.4 |
| 375 | 16038718 | -6.4 |
| 376 | 21679044 | -6.4 |
| 377 | 25154345 | -6.4 |
| 378 | 44393882 | -6.4 |
| 379 | 44437436 | -6.4 |
| 380 | 44575277 | -6.4 |
| 381 | 46907271 | -6.4 |
| 382 | 46907311 | -6.4 |
| 383 | 59741079 | -6.4 |
| 384 | 59876519 | -6.4 |
| 385 | 59876522 | -6.4 |
| 386 | 59897198 | -6.4 |
| 387 | 73353957 | -6.4 |
| 388 | 91034308 | -6.4 |
| 389 | 138968420 | -6.3 |
| 390 | 145069458 | -6.3 |
| 391 | 57403993 | -6.3 |
| 392 | 44393868 | -6.2 |
| 393 | 44393932 | -6.2 |
| 394 | 56776171 | -6.2 |
| 395 | 59876491 | -6.2 |
| 396 | 46907273 | -6 |
| 397 | 68169653 | -6 |
| 398 | 46906151 | -5.7 |
| 399 | 145970059 | -5.6 |
| 400 | 46907192 | -5.4 |
| 401 | 139258929 | -4.9 |
| 402 | 71589913 | -4.8 |
| 403 | 139258931 | -4.6 |
| 404 | 53436107 | -4.5 |
| 405 | 143607623 | -4.3 |
| 406 | 24178376 | -4.3 |
| 407 | 139258930 | -4.1 |
| 408 | 68796320 | -3.9 |
| 409 | 72194390 | -3.3 |
| 410 | 157746974 | -0.8 |
| 411 | 157887226 | -0.8 |
| 412 | 160619786 | -0.8 |
| 413 | 162237387 | -0.8 |


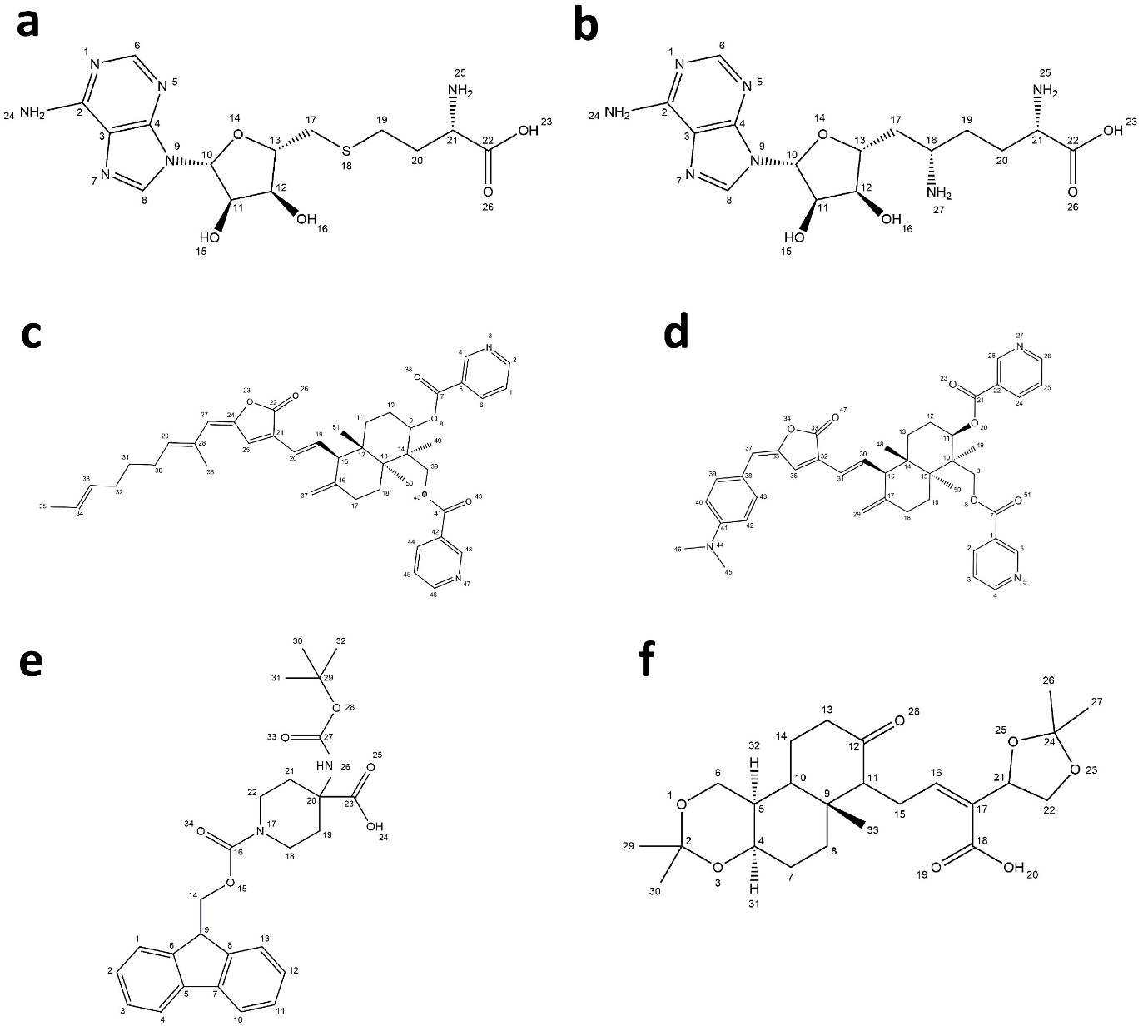


**Fig. S1.** 2D images showing atom numbering of the compounds (a) SAH, (b) SFG, (c) 44437487, (d) 44437491, (e) 2734589 and (f) 138968421.
